# Decreasing Lamin A Triggers Cell Fate Transitions through Heterochromatin-Nuclear Periphery Detethering

**DOI:** 10.34133/bmr.0256

**Published:** 2025-09-18

**Authors:** Lijuan Sun, Yafan Xie, Zhaoyan Zuo, Jian Liu, Jiaqi Yang, Iqra Ali, Qin Peng, Juhui Qiu

**Affiliations:** ^1^ Key Laboratory for Biorheological Science and Technology of Ministry of Education, State and Local Joint Engineering Laboratory for Vascular Implants, Bioengineering College, Chongqing University, Chongqing 400030, China.; ^2^ Institute of Systems and Physical Biology, Shenzhen Bay Laboratory, Shenzhen 518132, China.

## Abstract

The interplay between nuclear architecture and extracellular matrix stiffness orchestrates cell fate decisions, yet the molecular mechanisms remain poorly defined. Here, we investigate the role of Lamin A (*LMNA*), a nuclear structural protein whose expression correlates with tissue stiffness, in regulating cellular differentiation and fate decision. Using myoblasts and fibroblasts as models, it was observed that cells with low *LMNA* expression showed that higher cell deformation elevated expression of neurological genes and exhibited potential for differentiation into a neural-like fate. CUT&Tag sequencing of *LMNA*-knockdown cells revealed a reduction in the size of Lamin B1-associated domains, with enhanced Lamin B1 binding at muscle-related genes (*Myf5* and *Myf6*) and diminished binding at the neural gene *Nes*, suggesting that changes in gene expression are associated with alterations in chromatin structure. Further analysis identified the dissolution of H3K9me2/3-labeled heterochromatin regions and their redistribution in the nucleoplasm following *LMNA* inhibition. Soft substrates (0.2 kPa) amplify the neural differentiation capacity in *LMNA*-knockout cells. Additionally, retinoic acid was shown to enhance the expression of neurologically related genes by suppressing *LMNA* expression. These findings reveal a novel substrate stiffness-induced mechanism by which Lamin A regulates cell fate transitions and provide a new approach for neural cell generation.

## Introduction

The field of regenerative medicine increasingly relies on bioengineered materials to recapitulate physiological mechanical cues, because mechanical forces within the cell microenvironment can influence cell fate decisions [1,2]. Additionally, regulating intracellular mechanics with small molecules can also affect cell fate [3]. Cell fate transitions are contingent upon alterations in chromatin structure and gene transcription, with the influence of extracellular matrix forces and cytoskeletal dynamics depending on nuclear deformation mediated by the linker of nucleoskeleton and cytoskeleton complex and nuclear cytoskeleton [4,5]. The nuclear cytoskeletal lamina plays a regulatory role in cell fate at the cellular level and in preimplantation embryos [6,7]. Lamin A, a type of nuclear cytoskeleton, is integral to the shape, stiffness, and mechanical integrity of the nucleus [8,9]. The expression levels of Lamin A are closely correlated with nuclear stiffness, exhibiting higher levels in rigid tissue cells, such as bone and muscle, and lower levels in softer tissues, such as the liver, fat, and brain [10,11]. Notably, in contrast to normal cells, nuclei with mutations in *LMNA* (the gene encoding Lamin A) display increased nuclear area and reduced nuclear stiffness, along with non-myocyte gene expression in human induced pluripotent stem cell line - cardiomyocyte (hiPSC-CM) cells [12]. Conversely, matrix stiffness-driven contractility exerts tension on the nucleus, promoting Lamin A/C accumulation whereas suppressing soft tissue phenotypes [13,14]. These observations suggest that modulating the levels of Lamin A in cells could directly influence cell fate.

The interaction between the lamina and genome serves as a crucial regulator of gene expression [15,16]. As is well known, the dense heterochromatin layer that associates with the lamina is a hallmark feature located near the subnuclear periphery of most cells, characterized by low transcriptional activity [17]. For instance, heterochromatin located near the nuclear membrane may relocate to the interior of the nucleus during transient nuclear deformation, facilitating cell fate transitions through epigenetic modifications [18].

These heterochromatin regions, associated with Lamin A, are referred to as lamina-associated domains (LADs) and are enriched in heterochromatin-modifying markers such as H3K9me2, H3K9me3, and H3K27me3 [19]. Heterochromatin is typically anchored to the nuclear structure by LADs, which are plentiful in heterochromatin markers and exert inhibitory effects on gene expression [20]. Lamin A regulates gene expression by influencing gene localization within LADs, which may mediate tissue-specific gene silencing and activation [19]. Global changes in heterochromatin induced by Lamin A perturbations often manifest as altered levels of chromatin-associated epigenetic histone markers. The isolation or loss of heterochromatin has been documented in cells expressing various *LMNA* mutations, resulting in decreased levels of heterochromatin markers such as H3K9me3 and H3K27me3 [21,22]. Nevertheless, it remains unclear whether modulation of Lamin A expression in relation to cell fate is dependent on changes in LADs and alterations in heterochromatin modifications near the nuclear periphery.

Here, we demonstrate that inhibiting *LMNA* promotes the transdifferentiation of stiff cells, such as myoblasts and fibroblasts, into a neural-like fate. The underlying mechanism involves *LMNA* inhibition, which decreases chromatin aggregation and triggers the detethering of heterochromatin from the nuclear periphery. Soft substrates can amplify the neural differentiation capacity in *LMNA*-knockout (KO) cells. The *LMNA* inhibitor retinoic acid (RA) induces fibroblasts to acquire the potential for nerve cell fate, which may provide seed cells for neural tissue engineering and regenerative medicine.

## Materials and Methods

### Cell lines and cell culture

Mouse myoblasts and fibroblasts (C2C12 and NIH3T3) were purchased from the American Type Culture Collection (CRL-1772 and CRL-1658). KO cell lines were developed in our laboratory and cultured in Dulbecco’s Modified Eagle’s Medium supplemented with 10% fetal bovine serum (FBS) and 1% penicillin-streptomycin. Mouse neural stem cells (NE-4C) were purchased from the Cell Bank of the Chinese Academy of Sciences (SCSP-1501) and cultured in minimum essential medium supplemented with 10% FBS, 2 mM glutamine, and 1% penicillin-streptomycin solution (Invitrogen). Cell plates were treated with 0.01% poly-L-lysine (5 ml of 0.1% poly-L-lysine combined with 45 ml of ddH_2_O) at 37 °C for more than 2 h prior to cell recovery or passaging. All cells were maintained in a culture incubator at 37 °C with 5% CO_2_.

### CRISPR-mediated *LMNA* gene KO cell line

To KO *LMNA* genes, we first inserted the m*LMNA* gRNA into the pSpCas9(BB)-2A-GFP (PX458) (addgene#48138).

Two gRNAs were designed: gRNA1 (5′-GCGAGCTCCATGACCTGCGG-3′) and gRNA2 (5′-TCTCAGTGAGAAGCGCACAT-3′). These gRNAs were synthesized using the following primers: m*LMNA*-E2-gRNA1-F (5′-CACCGCGAGCTCCATGACCTGCGG-3′), m*LMNA*-E2-gRNA1-R (5′-AAACCCGCAGGTCATGGAGCTCGC-3′), m*LMNA*-E2-gRNA2-F (5′-CACCGTCTCAGTGAGAAGCGCACAT-3′), and m*LMNA*-E2-gRNA2-R (5′-AAACATGTGCGCTTCTCACTGAGAC-3′), and then cloned using the Golden Gate assembly method. Plasmids were validated by sequencing with LKO1-5 primers (5′-GACTATCATATGCTTACCGT-3′) using the Sanger sequencing method. The day prior to transfection, 0.4 million C2C12/NIH3T3 cells were seeded into a 6-well plate. A total of 1.25 μg of m*LMNA* 1-PX458-gRNA1 and 1.25 μg of m*LMNA* PX458-gRNA2 were cotransfected into C2C12/NIH3T3 cells using Lipofectamine 3000 Reagent. Forty-eight hours post-transfection, cells were subjected to fluorescence-activated cell sorting to isolate green fluorescent protein-positive single-cell clones into 96-well plates. After approximately 1 month of incubation, single clones were expanded and verified via genotyping PCR with Lamin A-specific primers (m*LMNA*-KOJC-F: 5′-TCGAGGCTCTTCTCAACTCC-3′ and m*LMNA*-KOJC-R: 5′-AGGTGAGCAGGCAAATGG) as well as Sanger sequencing.

### Fabrication of microfluidic device

**Fig. 1.**
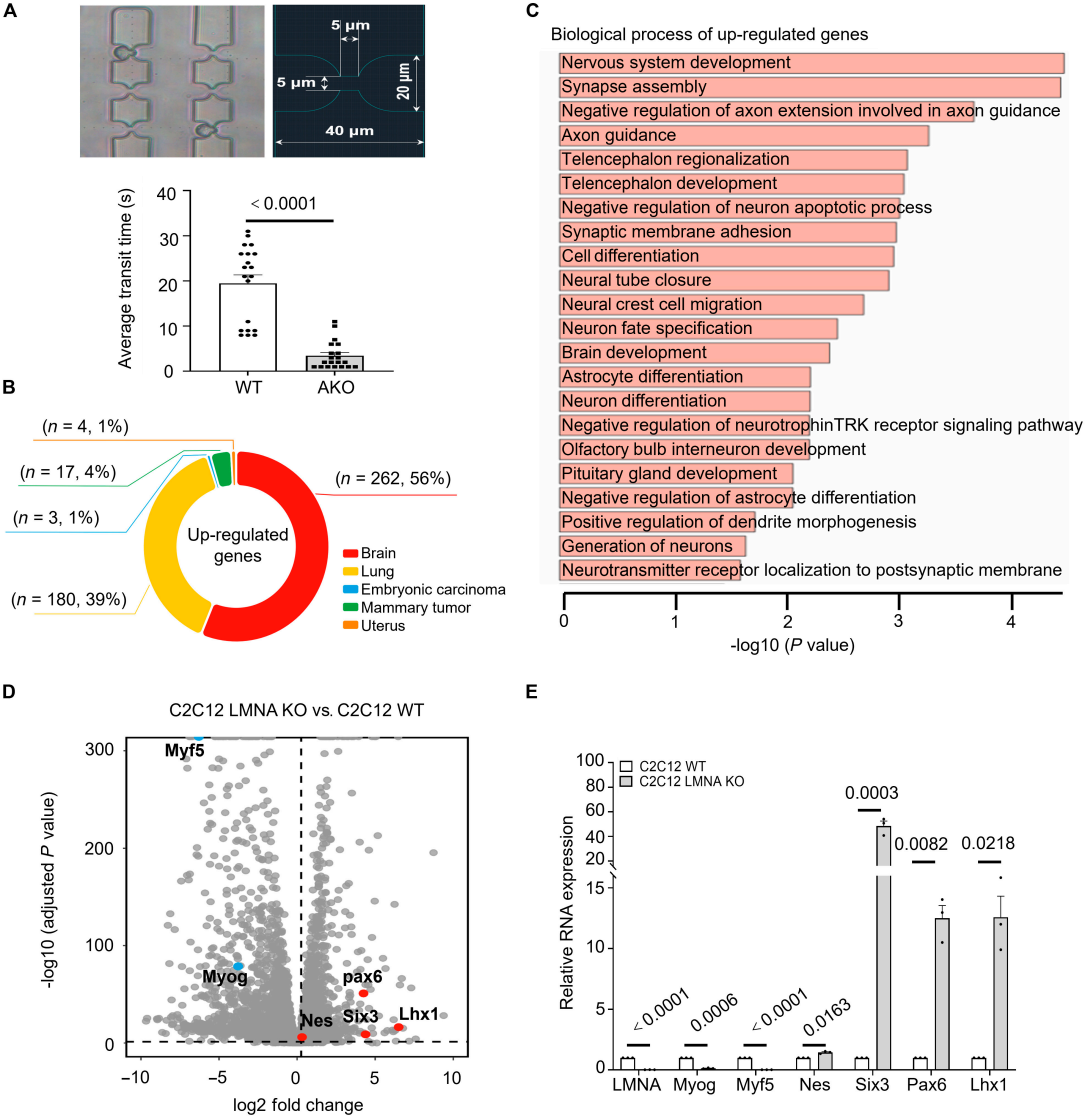
Knockout of *LMNA* in C2C12 has a neural-like fate. (A) A microfluidic device comprising small channels used for measuring cell stiffness. The top and left image depicts a cell suspension flowing through the microfluidic channel, with specific diameter measurements shown on the right. The bottom image shows time taken for a single cell to pass through the well. *n* = 20. (B) Ring plot illustrating the proportion of tissues enriched with up-regulated genes after *LMNA* KO. (C) GO analysis of up-regulated genes in C2C12 *LMNA* KO cells compared to C2C12. Only terms related to biological processes are displayed. (D) Volcano plot depicting differentially expressed genes in C2C12 *LMNA* KO cells compared to C2C12, where each point represents a gene. The dashed lines indicate the cutoff for significance, labeled as fold change > 1.2 and FDR < 0.05. Red points represent the notably up-regulated genes, such as *Nes*, *Pax6*, *Six3*, and *Lhx1*; blue points indicate down-regulated genes of interest, including *Myf5* and *Myog*. (E) qPCR quantification of the transcription levels of representative differentially expressed genes in both C2C12 and C2C12 *LMNA* KO cells.

The silicon master mold with micro-patterns was custom-fabricated by BYMICROFAB Corporation. As illustrated in Fig. 1A, the final polydimethylsiloxane (PDMS, Sylgard 184, Dow Corning) microstructure was achieved through 2 replication molding processes. The PDMS prepolymer was mixed with the curing agent at a 10:1 weight ratio and poured onto the silicon master to create a negative template. After degassing under vacuum for 30 min to remove air bubbles, the mixture was thermally cured at 60 °C for 3 h. The resultant negative PDMS mold was subsequently peeled off from the master, treated with oxygen plasma, and subjected to vapor-phase silanization with 3-(triethoxysilyl)-1-propanamine under vacuum overnight. For positive PDMS microstructure fabrication, the PDMS prepolymer was poured onto the negative PDMS template, followed by vacuum degassing for 30 min and overnight curing at 60 °C. The cured positive PDMS microstructure was carefully peeled from the negative template. Final device assembly involved treating both the positive PDMS microstructure and a clean glass coverslip with oxygen plasma (120 W, 90 s) to enable covalent bonding. The activated surfaces were immediately brought into conformal contact and bonded. The assembled microfluidic device was further cured at 60 °C for 3 h to enhance interfacial adhesion. Devices peeled from silicon substrates were bonded to coverslips using identical procedures.

Prior to loading, cells were trypsinized and filtered through a 40-μm cell strainer. The cell suspension was perfused through the micro-patterned channels at 0.002 ml/min using a microinjection pump. Cellular transit events through constriction pores were recorded in real-time via microscopy coupled with a charge-coupled device imaging system. The transit time, manually quantified from recorded videos, served as an indirect indicator of cellular stiffness, with prolonged transit durations correlating with increased mechanical rigidity.

### Preparation of polyacrylamide gels

Polyacrylamide (PAA) gels were prepared as described previously [23]. Briefly, tunable mechanical properties were prepared by varying the ratios of acrylamide to bis-acrylamide (Sangon Biotech, A100486 and A610508). The polymerization reaction was initiated using 10% ammonium persulfate and *N*,*N*,*N′*,*N′*-tetramethylethylenediamine as crosslinkers. The prepolymer solution was sandwiched between a glass slide and allowed to fully polymerize under ultraviolet light (365 nm) for 30 min. Then, the gels were coated with 20 μg/ml fibronectin overnight at 4 °C and 0.1% gelatin (Amresco, 9764) for 1 hour at 37 °C. After washing gels with PBST, cells were seeded on gels. Experiments were carried out 0.5 to 12 h after cell seeding.

### RA treatment

Cells were seeded at a density of 0.5 × 10^5^ per well in disposable 6-well cell culture plates and incubated for 12 h under standard culture conditions (37 °C, 5% CO₂). RA (10 μM in dimethyl sulfoxide [DMSO]; Sigma-Aldrich, R2625-50MG) was then administered for either 3 or 7 days. Transcriptional and protein-level analyses, along with immunofluorescence staining, were performed following the respective treatment durations. To maintain stable drug concentrations, the RA-supplemented medium was refreshed every 3 days based on the compound of half-life profile.

### Quantitative real-time PCR

The E.Z.N.A. Total RNA Kit I (Omega, R6834-01) was utilized to extract total RNA, following the manufacturer’s guidelines. For each reaction, 1 μg of RNA was reverse transcribed to cDNA using random primers and reverse transcriptase (Takara, RR036A). Quantitative real-time PCR (qPCR) was then performed using specific primers and Hieff qPCR SYBR Green Master Mix (Hieff, 11204ES08) in an MX3000P Stratagene PCR machine. The relative expression levels were normalized against the internal control, GAPDH. The following qPCR primers were used: GAPDH gene: Forward 5′-CAGAAGACTGTGGATGGCCC-3′ and Reverse 5′-ATCCACGACGGACACATTGG-3′; *LMNA* gene: Forward 5′-TGGCGGTAGAGGAAGTCGAT-3′ and Reverse 5′-CTTCGGTGGGAAGCGATAGG-3′; *Myog* gene: Forward 5′-CAGCCCAGCGAGGGAATTTA-3′ and Reverse 5′-AGAAGCTCCTGAGTTTGCCC-3′; *Myf5* gene: Forward 5′-CGGATCACGTCTACAGAGCC-3′ and Reverse 5′-GCAGGAGTGATCATCGGGAG-3′; *Nes* gene: Forward 5′-GGTAGGGCTAGAGGACCCAA-3′ and Reverse 5′-AGCCCTTGCATTCCAGAGTC-3′; *Pax6* gene: Forward 5′-GGGCAATCGGAGGGAGTAAG-3′ and Reverse 5′-GCTTTTCGCTAGCCAGGTTG-3′; *Lhx1* gene: Forward 5′-ATTCCGAGCTATCCCCACCT-3′ and Reverse 5′-CTAGGAAGGTCTCGGTCCCA-3′; and *Six3* gene: Forward 5′-ACGCCAACAGACAGTCACAA-3′ and Reverse 5′-CCGTGGGAACCAAAAGGAGA-3′.

### Western blot

Cells were lysed using RIPA buffer, and protein samples were separated by 10% TGX stain-free PAA gels before being transferred to 0.45-μm PVDF membranes. The membranes were blocked with 5% skimmed milk/PBST for 2 h at room temperature, followed by incubation with primary antibodies overnight at 4 °C. Afterward, the membranes were incubated with horseradish peroxidase (HRP)-conjugated secondary antibodies for 1 h at room temperature. HRP was detected using ECL Western Blotting Substrate (Epizyme, SQ201), and images were captured using the ChemiDoc MP Imaging System (Bio-Rad). The intensity of the bands was analyzed with ImageJ software. The following antibodies were used: GAPDH (CST 2118, 1:5,000), Nestin (Abcam ab221660, 1:1,500), and Pax6 (CST 60433, 1:200).

### Immunofluorescence staining

For immunofluorescence staining, 0.1 million C2C12 cells were seeded on a 15-mm dish. The cells were fixed with 4% paraformaldehyde (Biosharp, BL539A) for 30 min at room temperature, followed by permeabilization with 0.25% Triton X-100 (Sangon Biotech, A110694) for 30 min. The cells were then blocked with 5% bovine serum albumin/PBST for 1 h and incubated with primary antibodies overnight at 4 °C. Subsequently, the cells were washed 3 times with PBST for 10 min each. The gels or dishes were then incubated with secondary antibodies at room temperature for 1 h, and the cells were stained with Hoechst 33342 before undergoing another wash with PBST. The gels were mounted with VECTASHIELD Antifade Mounting Medium (Vectorlabs, H-1000). The following antibodies were used: Nestin (Abcam ab221660, 1:500), Pax6 (CST 60433, 1:200), H3K9me2 (Active Motif 39239, 1:500), H3K9me3 (CST 13969S, 1:200), H3K9ac (CST 9649, 1:500), and H3K27me3 (Active Motif 39155, 1:500).

### RNA-seq data processing

Total RNA was extracted from C2C12 and C2C12 *LMNA* KO cells using the E.Z.N.A. Total RNA Kit I (Omega). A minimum of 3 biological replicates were analyzed. The RNA samples were submitted to Geneplus for library preparation and sequencing, which was conducted on the DNBSEQ-T7 platform with 150 bp paired-end reads.

Raw RNA reads were trimmed using Ktrim (version 1.3.0) software to remove adapters and filter out low-quality reads. Clean reads were subsequently aligned to the UCSC mouse mm10 reference genome using STAR (version 2.7.9a). Expression quantification was carried out with the featureCounts tool from the subread package (version 2.0.0). Differentially expressed genes (DEGs) were identified using DESeq2 (version 1.32.0), applying a cutoff of fold change > 1.2 and adjusted *P* value < 0.05 to filter differentially regulated genes. Functional annotation and tissue enrichment analyses were performed using the DAVID website.

### CUT&Tag library preparation and sequencing

For CUT&Tag library preparation and sequencing, Lamin B1 (Abcam ab16048) was examined in C2C12 and C2C12 *LMNA* KO cells using the Hieff NGS In-Situ DNA Binding Profiling Library Prep Kit for Illumina (Yeason), following the manufacturer’s instructions. DNA libraries were constructed in accordance with the manufacturer’s protocol and sequenced on the NovaSeq X Plus platform with paired-end reads of 150 bp.

Raw DNA reads were processed using Ktrim (version 1.3.0) software to remove adapters, followed by alignment to the UCSC mouse mm10 reference genome using Bowtie2 (version 2.3.5.1). SAM files were filtered with Samtools (version 1.5), converted to BAM format, and sorted. Duplicate reads were eliminated using Picard (version 2.18.29), and the resulting files were converted to BED format with Bedtools (version 2.26). Peak calling was then performed using SEACR (version 1.3). BigWig tracks were generated using bamCompare and bamCoverage from deepTools (version 3.1.1). The ComputeMatrix and plotHeatmap functions were utilized to create a heatmap of CUT&Tag signals at transcription units. LADs were identified using EDD (version 1.1.18) with automatic estimation of GapPenalty and BinSize. Peak files and BAM files were analyzed using DiffBind (version 3.14) for differential binding analysis. Each peak was annotated utilizing ChIPseeker (version 1.40).

## Results

### *LMNA* inhibition enhances neural related gene expression

To investigate whether *LMNA* levels influence gene expression, an *LMNA* KO cell line was constructed based on mouse myoblasts (C2C12) using the CRISPR/Cas9 system (Fig. S1). Following the KO of *LMNA*, the nuclei of C2C12 cells were significantly enlarged, and the aspect ratio of the cells was markedly increased (Fig. S2). Cellular deformation was assessed using a microfluidic loading device. The results showed that, although the nuclear area of wild-type (WT) cells was smaller, C2C12 WT cells traversed the microfluidic channel more slowly than the *LMNA* KO cells (Fig. 1A), indicating that *LMNA* KO cells exhibit greater deformability.

To elucidate the potential mechanism underlying the increased softness of *LMNA* KO cells, RNA sequencing was conducted using total RNA extracted from both C2C12 WT and C2C12 *LMNA* KO cells. The results revealed that the KO of *LMNA* in C2C12 affects gene expression, with a total of 1,883 significantly DEGs identified compared to C2C12 WT cells. Of these, 1,243 genes were down-regulated, and 640 genes were up-regulated. The up-regulated genes were predominantly enriched in brain tissue (Fig. 1B), whereas the expression of muscle-related genes was significantly down-regulated (Fig. S3).

Subsequently, we performed gene ontology (GO) enrichment analysis on the up-regulated genes following *LMNA* KO in C2C12 to investigate their functions. Results demonstrated that 16% of the biological process terms enriched by these up-regulated genes were directly related to neurodevelopment, including synaptic assembly, axon guidance, nervous system development, and neuronal differentiation (Fig. 1C). The genes enriched in each term are detailed in Table S1. These findings suggest that the knockdown of *LMNA* in C2C12 cells can enhance their potential for differentiation into neural-like fate cells.

Myog and Myf5 are transcription factors that determine muscle fate. Myf5 initiates the differentiation of myoblasts, whereas Myog is highly expressed during the late stage of myoblast differentiation to trigger cell fusion and myotube formation [24–27]. Nestin is a neuroepithelial stem cell protein and a cytoskeletal intermediate filament, originally characterized in neural stem cells [28–31]. The transcription factors Pax6, Lhx1, and Six3 are mainly enriched in cells of the neurodevelopmental system [32–34]. The results showed that *Myog* and *Myf5* expression were significantly down-regulated, whereas *Nes*, *Pax6*, *Lhx1*, and *Six3* expression were significantly up-regulated following *LMNA* KO (Fig. 1D and E). These findings suggest that, after *LMNA* KO, myogenic transcription factors in C2C12 cells were down-regulated, whereas nerve-related genes were up-regulated, indicating a shift in cell fate toward neurogenic differentiation.

### *LMNA* inhibition enhances cell obtain neural-like fate

**Fig. 2.**
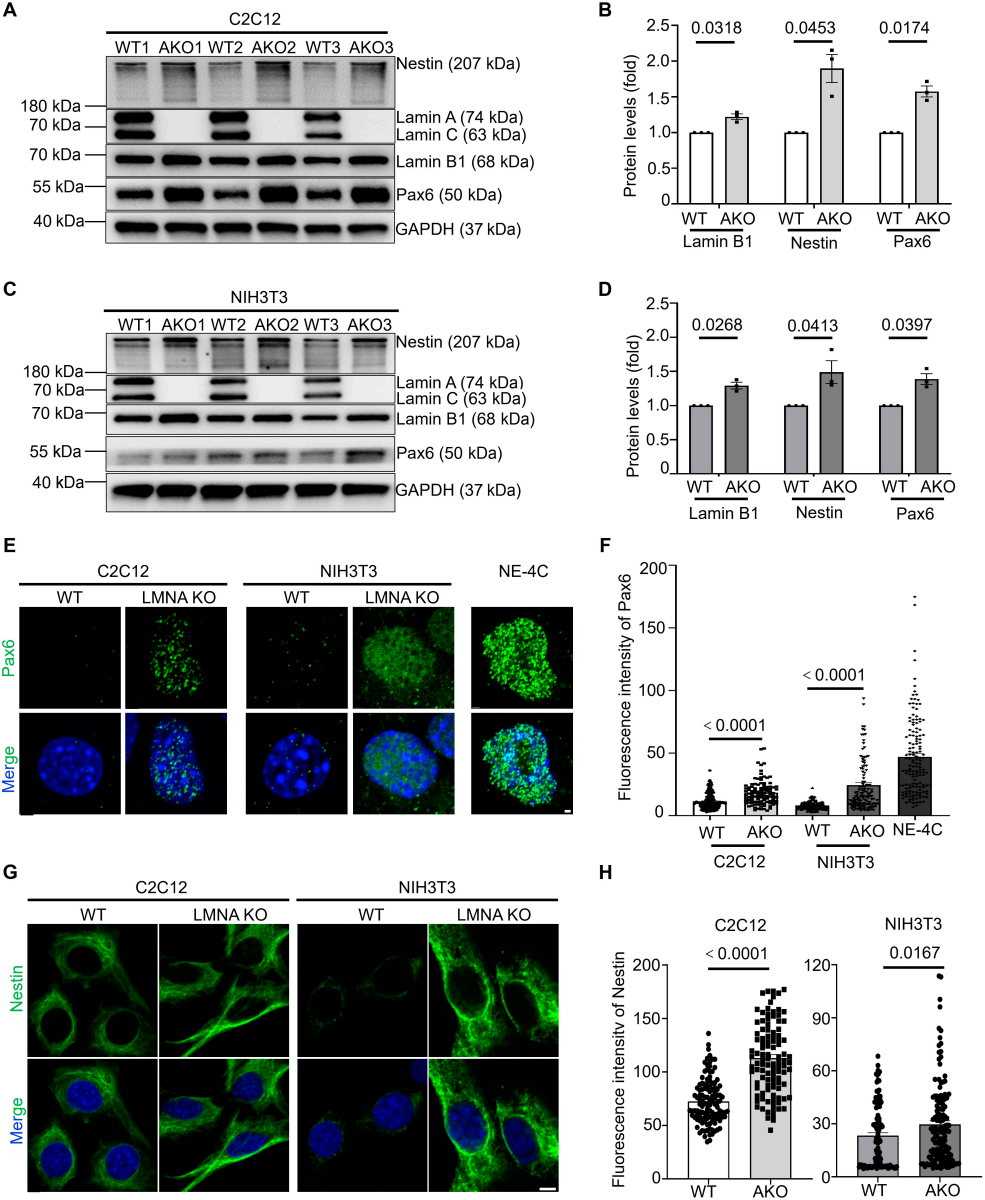
High expression of neural-related genes in *LMNA* KO cells. (A) WB analysis of Nestin and Pax6 in C2C12 WT and C2C12 *LMNA* KO cells. (B) Quantitative analysis of protein expression in C2C12 WT and C2C12 *LMNA* KO (data presented as mean ± SEM, *n* = 3 biological replicates, with using GAPDH as a loading control, statistical analysis using 2-tailed Student’s *t* test). (C) WB analysis of Nestin and Pax6 in NIH3T3 WT and NIH3T3 *LMNA* KO cells. (D) Quantitative analysis of differential protein levels in NIH3T3 WT and NIH3T3 *LMNA* KO cells. (E) Visualization of Pax6 expression and localization in WT and *LMNA* KO cells, with NE-4C included as a positive control. Scale bar: 2 μm. (F) Mean fluorescence intensity of Pax6 quantified, with *n* > 100. Pax6 mean fluorescence intensity greater than 20 was defined as positive, and less than 20 was designated as negative. (G) Visualization of Nestin expression and localization in WT and *LMNA* KO cells. Scale bars: 10 μm. (H) Quantification of mean fluorescence intensity of Nestin. *n* > 100.

To further validate the effect of *LMNA* KO on cell fate transition, mouse fibroblasts (NIH3T3) were employed. The nucleus of *LMNA* KO NIH3T3 cells exhibited enlargement and facilitated faster passage through the microfluidic channel compared to WT cells (Fig. S4). Western blot (WB) analyses confirmed a significant increase in the expression of Nestin and Pax6 following *LMNA* KO in both C2C12 and NIH3T3 cells (Fig. 2A to D).

To further investigate the effect of *LMNA* on cell fate transitions, Pax6 staining was performed on both NIH3T3 and C2C12 cell lines, using the neural stem cell line NE-4C as a control. The results demonstrated that Pax6 expression was nearly absent in WT cells, whereas a significant increase in nuclear Pax6 expression was observed in *LMNA* KO cells, aligning with the expression levels in NE-4C (Fig. 2E and F). Statistical analyses indicated that the proportion of Pax6-positive cells markedly increased in both C2C12 and NIH3T3 cells following *LMNA* KO compared to WT cells; However, a subset of Pax6-negative cells remained present (Fig. S5), suggesting that the KO of *LMNA* does not fully induce all cells to transdifferentiate into neuronal cells. Similarly, the distribution of Nestin showed a significant increase in average fluorescence intensity in both C2C12 and NIH3T3 *LMNA* KO cells compared to WT cells (Fig. 2G and H).

### KO *LMNA* decreases the size of LADs

**Fig. 3.**
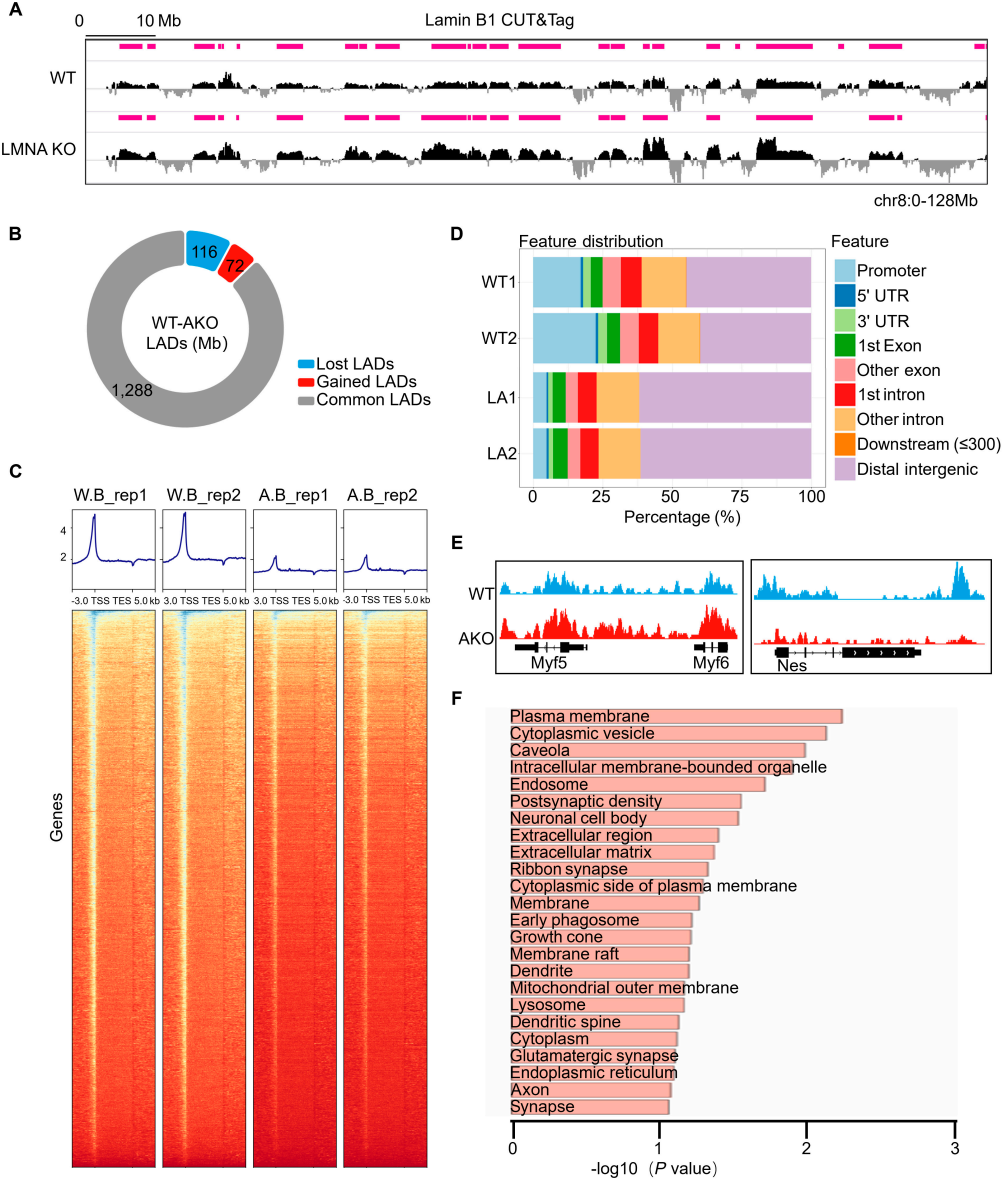
Dynamic features in LADs after KO of *LMNA*. (A) Genome browser view of the Lamin B1 CUT&Tag signal [log2(Lamin B1/IgG)] in the region of chromosome 8 for C2C12 and C2C12 *LMNA* KO cells. (B) Ring plot illustrating alterations in genomic coverage of LADs post-*LMNA* KO. (C) Heatmap showing Lamin B1 signals over transcription units, plotted for the region extending 3 kb upstream of the transcription start site (TSS) to 3 kb downstream of the transcription end site (TES) in C2C12 and C2C12 *LMNA* KO cells. (D) Distribution of genomic features on LADs in C2C12 and C2C12 *LMNA* KO cells. (E) Representative tracks displaying Lamin B1 signals at *Myf5*, *Myf6*, and *Nes* genes in C2C12 and C2C12 *LMNA* KO cells. (F) GO analysis of genes with up-regulated expression and reduced Lamin B1 binding affinity in C2C12 *LMNA* KO cells compared to C2C12.

Evidence suggests that Lamin A/C regulates gene expression through LADs [35], whereas Lamin B1 serves as the primary binding protein for LAD detection via the CUT&Tag assay [36,37]. Our analysis revealed a significant 116-Mb loss of LADs from Lamin B1 CUT&Tag data (Fig. 3A), which exceeded the gains observed following the KO of *LMNA* (Fig. 3B). Further examination showed that Lamin B1 on the transcription units decreased after *LMNA* KO (Fig. 3C). Specifically, gene feature annotation of LADs demonstrated that promoter regions comprised 21.94% and 23.39% of the total LAD regions in the 2 groups of C2C12 WT cells, whereas in the 2 groups of C2C12 *LMNA* KO, the promoter regions constituted only 5.37% and 5.54% (Fig. 3D). These findings suggest that these promoter regions may be released, thereby facilitating the expression of neuron-related genes. Concurrently, we observed that muscle-specific transcription factors *Myf5* and *Myf6* in C2C12 cells exhibited increased binding to Lamin B1, whereas the neuron-related gene *Nes* demonstrated decreased binding to Lamin B1 post-*LMNA* KO (Fig. 3E). This trend appears to be inversely correlated with gene expression changes, indicating an association between gene expression and shifts in LADs.

To further elucidate the connection between changes in gene expression in C2C12 cells following *LMNA* KO and chromatin structure alterations, we integrated CUT&Tag and RNA-seq data for analysis. There are 3,362 and 593 significantly altered genes in the CUT&Tag and RNA seq datasets, respectively, of which 311 genes are shared by both. Notably, among these, 99 genes were up-regulated and exhibited reduced Lamin B1 binding affinity in C2C12 *LMNA* KO cells compared to C2C12 WT cells (Fig. S6). These results suggest that changes in gene expression are, to some degree, associated with alterations in LADs. Subsequently, GO enrichment analysis conducted on this subset of genes highlighted that the enriched terms were predominantly related to neuronal cellular components, including neuronal cell body, synapse, axon, and dendrite (Fig. 3F). Therefore, the KO of *LMNA* leads to a decrease in LAD size and the release of neural-related genes.

### *LMNA* KO alters the distribution of histone methylation modifications

**Fig. 4.**
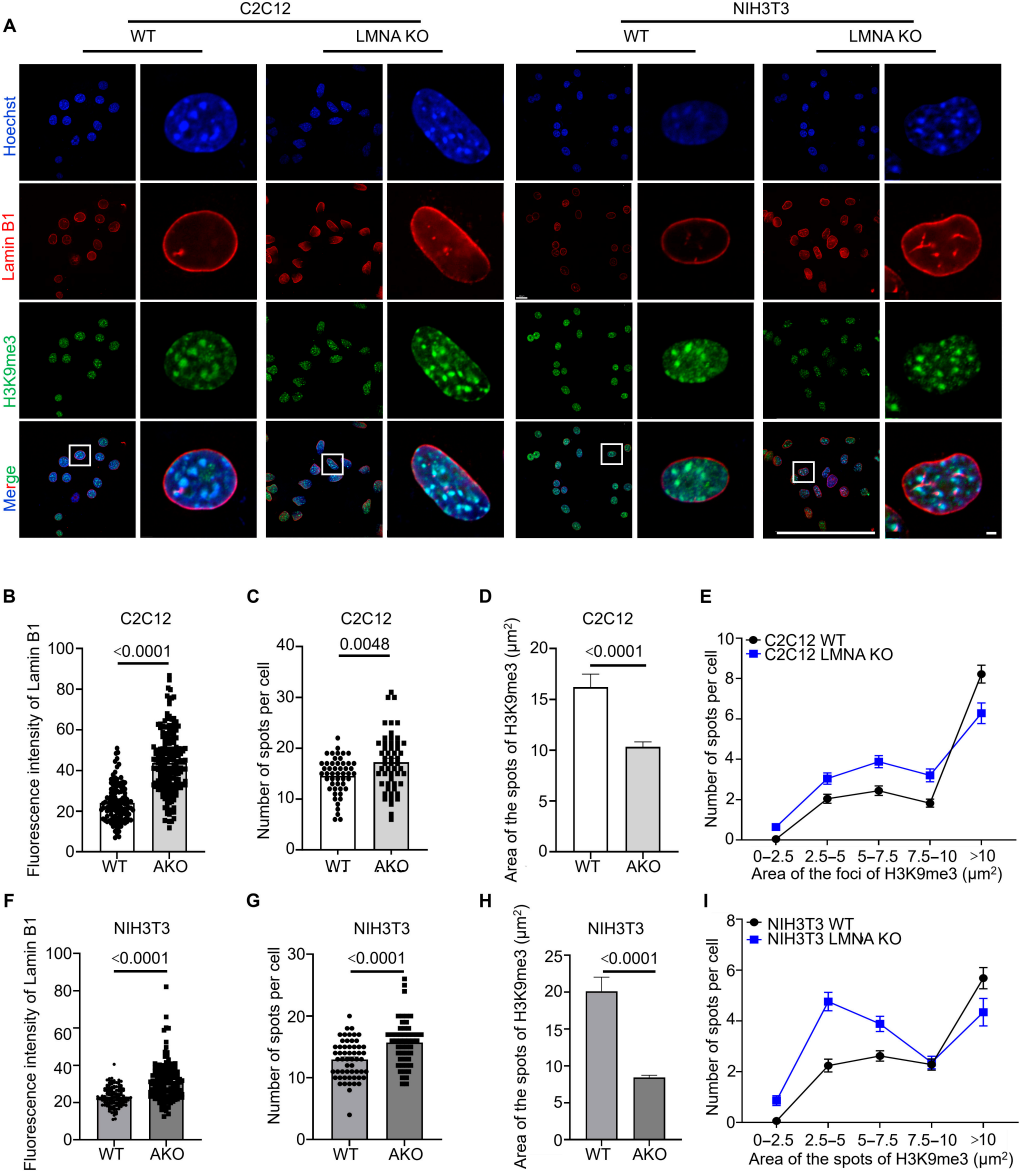
KO of *LMNA* in C2C12 alters the spatial distribution of histone methylation modifications. (A) Immunofluorescence images of Lamin B1 and H3K9me3 in cells following *LMNA* KO. Scale bars: 3 μm. (B) Quantification of the mean fluorescence intensity of Lamin B1 in WT and C2C12 *LMNA* KO. *n* = 126 and 191. (C) Number of H3K9me3 spots per cell for WT and C2C12 *LMNA* KO. *n* = 50 and 53. (D) Area of H3K9me3 spots in WT and C2C12 *LMNA* KO. *n* = 344 and 438. (E) Area distribution of H3K9me3 spots in each cell of WT and C2C12 *LMNA* KO. *n* = 50. (F) Quantitative analysis of the mean fluorescence intensity of Lamin B1 in WT and NIH3T3 *LMNA* KO. *n* = 126 and 142. (G) Number of H3K9me3 spots per cell for WT and NIH3T3 *LMNA* KO. *n* = 56 and 50. (H) Area of H3K9me3 spots in WT and NIH3T3 *LMNA* KO. *n* = 359 and 395. (I) Area distribution of H3K9me3 spots in each cell of WT and NIH3T3 *LMNA* KO. *n* = 50.

LADs and chromatin distribution are complexly associated with epigenetic modification [38,39]. Following *LMNA* KO, WB analysis revealed no significant changes in the total levels of H3K9me2 and H3K9me3 in the KO cells (Fig. S7). The spatial distribution of H3K9me3 was examined using immunofluorescence techniques (Fig. 4A). The number of H3K9me3 spots per cell significantly increased (Fig. 4C and G). To quantify the area of the H3K9me3 spots, a spot diameter of 1 μm was used. The analysis showed that the area of H3K9me3 spots in both C2C12 *LMNA* KO and NIH3T3 *LMNA* KO cells was smaller compared to their WT counterparts (Fig. 4D and H). Further analysis of the area distribution of H3K9me3 spots in each cell, using a 2.5-μm^2^ range, indicated that the size of the spots decreased to less than 10 μm^2^ following *LMNA* KO (Fig. 4E and I). These results suggest that *LMNA* disruption leads to the depolymerization of H3K9me3 spots, forming numerous smaller spots. Additionally, the average fluorescence intensity of Lamin B1 was higher in *LMNA* KO cells compared to WT cells (Fig. 4B and F), although only portions of the nuclear membranes showed Lamin B1 coverage. These findings suggest that dense heterochromatin regions may begin to dissolve following *LMNA* KO, facilitating the release of tethered genes and promoting transcriptional activity.

**Fig. 5.**
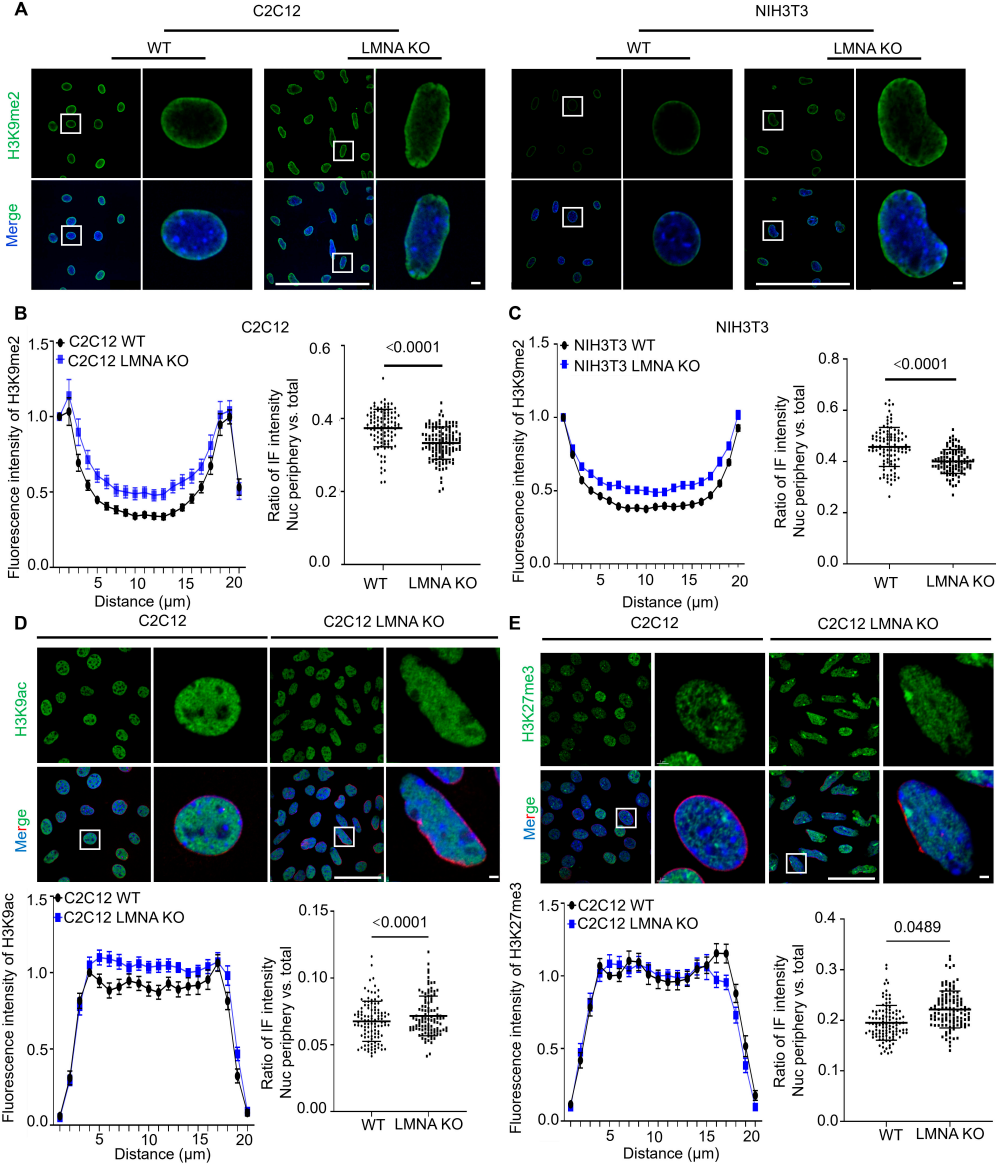
Increased nucleoplasmic distribution of H3K9me2 in C2C12 after KO of *LMNA*. (A) Immunofluorescence image of H3K9me2 in cells post-*LMNA* KO. Scale bars: 3 μm. (B) The distribution of H3K9me2 from perinuclear to nucleoplasm in C2C12 cells after *LMNA* KO was quantitatively analyzed, with the *x*-axis representing 20 equal divisions from perinuclear to nucleoplasm, and the perinuclear fluorescence intensity set to 1. *n* = 100. (C) Quantitative analysis of the distribution of H3K9me2 from perinuclear to nucleoplasm in NIH3T3 cells following *LMNA* KO. *n* = 100. (D) Decreased nucleoplasmic distribution of H3K9ac after *LMNA* KO in C2C12, based on data from 100 independent cells, presented as mean ± SEM, 2-tailed *t* test, *P* < 0.0001. Red is Lamin B1. Scale bars: 3 μm. (E) Decreased nucleoplasmic distribution of H3K27me3 post-*LMNA* KO in C2C12, based on data from 100 independent cells, presented as mean ± SEM, 2-tailed *t* test. Red is Lamin B1. Scale bars: 3 μm.

Furthermore, histone modifications H3K9me2, H3K27me3, and H3K9ac were stained (Fig. 5A, D, and E) and analyzed by dividing each cell into 20 equal parts along a “line” drawn from one end of the cell to the other, from the perinuclear region to the nucleoplasm. The average fluorescence intensity of each histone modification was then measured in each aliquot. The analysis revealed that H3K9me2 was significantly enriched around the nucleus in both C2C12 WT and NIH3T3 WT cells, whereas its nucleoplasmic distribution was significantly increased in C2C12 *LMNA* KO and NIH3T3 *LMNA* KO cells (Fig. 5B and C). In contrast, the nucleoplasmic distribution of H3K27me3 and H3K9ac was reduced in both C2C12 *LMNA* KO and NIH3T3 *LMNA* KO cells (Fig. 5D and E), and WB analysis revealed no significant changes in the total levels of H3K27me3 in the KO cells (Fig. S8). These results suggest that *LMNA* KO disrupts perinuclear chromatin, leading to the dissolution of perinuclear heterochromatin regions, which may facilitate the restoration of gene transcription in these areas and enhance gene expression.

### Soft substrates amplify the neural differentiation capacity in *LMNA*-KO cells

**Fig. 6.**
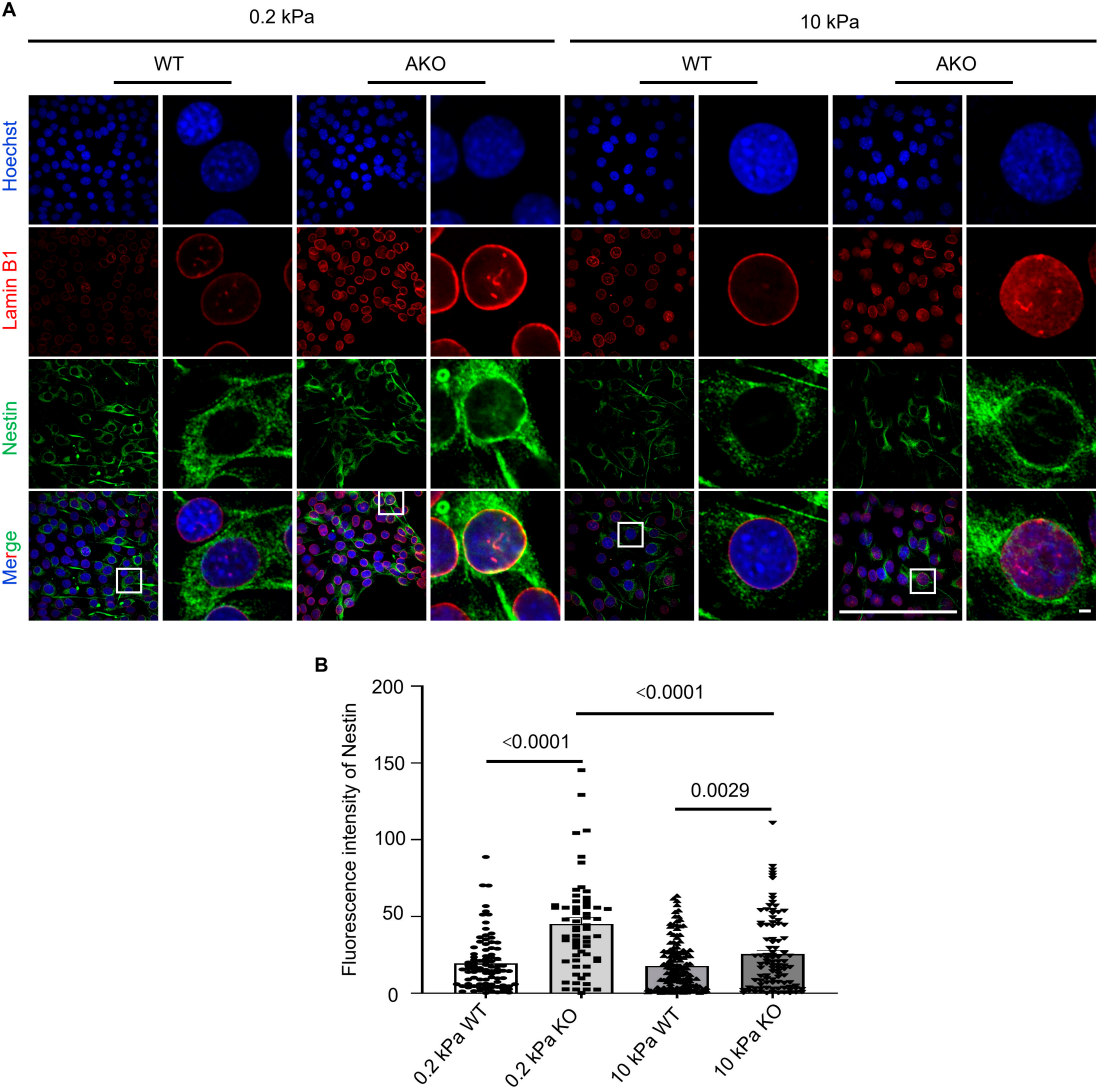
Soft matrix up-regulates the expression of Nestin for *LMNA* KO cells. (A). Visualization of Nestin expression and localization in WT and *LMNA* KO cells on 0.2 and 10 kPa. Scale bars: 3 μm. (B). Quantification of mean fluorescence intensity of Nestin. *n* > 100, 2-tailed *t* test.

Given that soft substrates down-regulate *LMNA* expression and connect with the neural fate [11], we hypothesized that matrix stiffness could modulate *LMNA*’s role in cell fate decisions. To test this, we cultured NIH3T3 and *LMNA*-KO NIH3T3 cells on fibronectin/gelatin-coated PAA hydrogels with stiffnesses of 0.2 kPa (soft, mimicking brain tissue) and 10 kPa (stiff, resembling muscle tissue). Quantitative immunofluorescence analysis revealed that *LMNA* KO cells exhibited significantly higher Nestin expression on 0.2 kPa substrates compared to both 10 kPa substrates and WT cells under the same mechanical conditions (Fig. 6A and B). These findings establish that soft matrix stiffness acts as a costimulus to potentiate the neural differentiation capacity in *LMNA*-KO cells. Mechanistically, this mechanical amplification likely occurs through nucleus deformation-driven chromatin remodeling, as observed in our previous studies of LADs reorganization (Fig. 3). Such substrate-engineered crosstalk between mechanical and genetic pathways offers a promising strategy for biomaterial-based neural induction.

### Inhibition of *LMNA* with RA promotes fibroblast to neural differentiation

To optimize clinical applications, small-molecule drugs that inhibit the expression of *LMNA* show promise as treatments for generating neural cells. RA serves as an inhibitor of *LMNA* expression by modulating the nuclear localization of RA transcription factors [11].

**Fig. 7.**
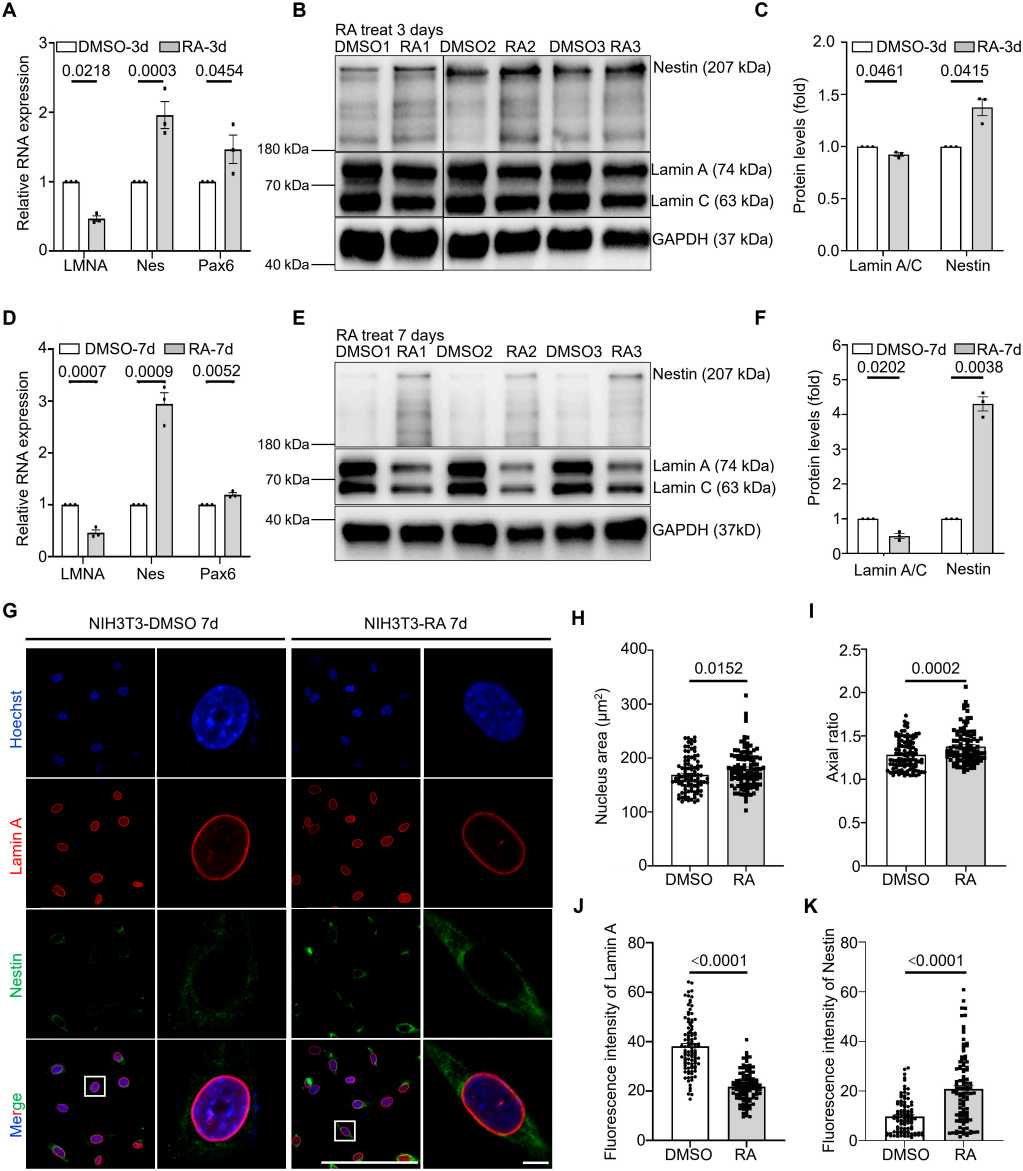
Inhibition of *LMNA* expression by retinoic acid up-regulates the expression of neural-related genes. (A) qPCR quantification of *LMNA*, *Nes*, and *Pax6* in NIH3T3 cells after 3 days of retinoic acid treatment. (B) WB analysis of related proteins in NIH3T3 cells after 3 days of retinoic acid treatment. (C) Quantitative analysis of related proteins in NIH3T3 cells after 3 days of retinoic acid treatment. (D) qPCR quantification of *LMNA*, *Nes*, and *Pax6* in NIH3T3 cells after 7 days of retinoic acid treatment. (E) WB analysis of related proteins in NIH3T3 cells following 7 days of retinoic acid treatment. (F) Quantification of related proteins in NIH3T3 cells after 7 days of retinoic acid treatment. All experiments were conducted with 3 replicates. (G) Immunofluorescence images of NIH3T3-DMSO 7d and NIH3T3-RA 7d cells. Scale bar: 3 μm. (H) Quantitative analysis of nucleus area for NIH3T3-DMSO 7d and NIH3T3-RA 7d. *n* = 97 and 113. (I) Quantitative analysis of the ratio of the long and short axes of NIH3T3-DMSO 7d and NIH3T3-RA 7d. *n* = 105 and 101. (J) Quantitative analysis of the mean fluorescence intensity of Lamin A. *n* = 101 and 107. (K) Quantification of the mean fluorescence intensity of Nestin. *n* = 98 and 102.

NIH3T3 cells were treated with RA for 3 and 7 days, after which the expression levels of *LMNA* and the neural marker genes *Nes* and *Pax6* were analyzed using qPCR and WB. The results indicated a significant reduction in both the transcription and protein levels of Lamin A following 3 and 7 days of RA treatment, with a more pronounced effect observed after 7 days, demonstrating that RA indeed inhibits the expression of *LMNA*. Furthermore, the transcription levels of *Nes* and *Pax6* in NIH3T3 cells were significantly elevated following *LMNA* inhibition, and the protein levels of Nestin also showed a notable increase (Fig. 7A to F). Morphologically, NIH3T3 nuclei were significantly enlarged, and the cells exhibited an elongated and narrow shape, with a marked enhancement in the distribution of Nestin and a significant increase in its average fluorescence intensity following *LMNA* inhibition (Fig. 7G to K). This observation aligns with the characteristics of the *LMNA* KO group. Consequently, the inhibition of *LMNA* in NIH3T3 cells induced by RA results in the elevated expression of neurodevelopmentally associated genes, *Nes* and *Pax6*.

## Discussion

**Fig. 8.**
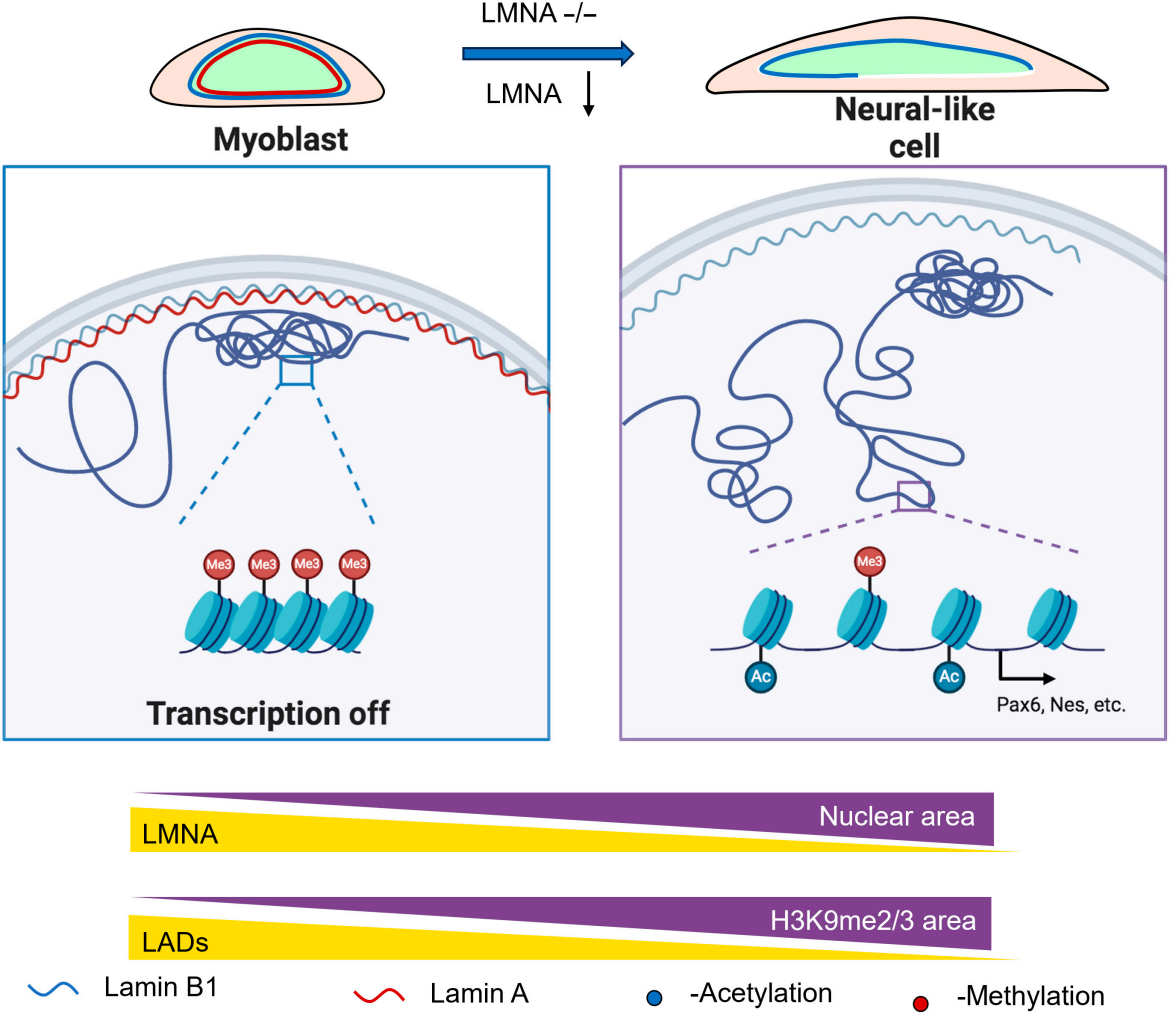
The summary of decreasing lamin A triggers cell fate transitions.

The aim of this study was to investigate the roles of *LMNA* in cell fate determination and the underlying mechanisms. We have characterized how low *LMNA* levels prime the transition to a neural-like cell fate by modulating chromatin redistribution and epigenetic modifications. Our study has produced the following findings: (a) KO or knockdown of *LMNA* enhances the acquisition of a neural-like fate in cells; (b) knocking out *LMNA* decreases the size of LADs and associates the *Myog* gene with LADs through histone modification; and (c) RA promotes neural-like fate in fibroblast cells by inhibiting *LMNA* expression (Fig. 8). Thus, our findings reveal a novel function of *LMNA*, demonstrating that it regulates cell fate through epigenetic modification and chromatin reorganization.

Lamin A maintains the integrity of the nucleus. In this study, we observed that the nuclear area increased in C2C12 and NIH3T3 cells following the KO of *LMNA*, leading to cell elongation and a decrease in cell stiffness, consistent with previous studies [11]. For instance, the absence of Lamin A in mouse fibroblasts results in enlarged nuclei and reduced cell stiffness [9]. Similarly, enlarged nuclei were also observed in cardiomyocytes derived from a heterozygous T10I Lamin A mutant hiPSC [12]. A recent study demonstrated that the actomyosin contractility inhibitor blebbistatin can induce the transdifferentiation of human foreskin fibroblasts into neurons, resulting in reduced cell stiffness during this process [3]. Moreover, the expression levels of neural cell marker genes *Nes* and *Pax6* in C2C12 and NIH3T3 cells markedly increased following *LMNA* KO. Nestin is a neuroepithelial stem cell protein, whereas Pax6 is a multifunctional transcription factor that regulates the proliferation and differentiation of neural stem cells. Lhx1 also plays a role in neurogenesis [31–33]. These results suggest that *LMNA* KO cells may exhibit characteristics akin to neural stem-like cells. Therefore, KO of *LMNA* promotes high expression of neurologically associated genes in C2C12 and NIH3T3 cells without exhibiting neuronal morphology, which may be due to the absence of culture using medium that induces neural differentiation; that is, with the addition of N2, B27, and human basic fibroblast growth factor 2, the medium can induce neuronal cell morphology [3]. Consequently, the effect of *LMNA* KO on cell fate concerning neurogenesis warrants further investigation.

Lamin A plays a critical role in regulating gene expression by influencing gene localization within LADs. In contrast, heterochromatin is anchored perinuclearly through LADs and exerts inhibitory effects on gene expression [19,21]. Results from our study indicate that the KO of *LMNA* in C2C12 and NIH3T3 cells did not affect the levels of H3K9me3 and H3K9me2. This observation is inconsistent with reports demonstrating that in C2C12 myoblasts with the R453W mutation of Lamin A, the modification of H3K9me3 and heterochromatin is markedly diminished [40]. A possible explanation for this discrepancy is that Lamin A interacts with DNA via its carboxy-terminal domain, resulting in differing functionalities between mutant and KO cells. Notably, after *LMNA* KO, the number of H3K9me3 spots in C2C12 cells increased whereas the area of these spots decreased, suggesting that the heterochromatin regions labeled by H3K9me3 became less compact, thereby making chromatin more open and facilitating the transition of cells into neuronal phenotypes. Supporting this finding, previous research has reported the loss of perinuclear heterochromatin to the nucleoplasm in the absence of Lamin A [41].

Our results also reveal that the expression of the myogenic transcription factor *Myf5* is reduced in cells following *LMNA* KO, whereas up-regulated genes are predominantly enriched in terms related to neurological functions. This alteration is primarily attributed to changes in the structure of Lamin B1 upon Lamin A deletion, which subsequently modifies the structure of LADs and affects the binding affinity of related genes to Lamin B1 [42]. Lamin B1 is mainly localized perinuclearly and is predominantly associated with transcriptionally inactive chromatin. In contrast, Lamin A/C is distributed in both the perinuclear area and nucleoplasm, interacting with both heterochromatin and euchromatin [37,43].

It is worth noting that Lamin A is required for neural commitment of sarcoma cells [44]. Thus, it appears that Lamin A is necessary even for that differentiation process, although a very low amount could be required. To circumvent the all-or-none effects of complete *LMNA* KO, we subsequently employed several strategies to reduce *LMNA* expression. RA has been shown to not only inhibit *LMNA* expression in some cell types [11] but also increase *LMNA* expression [45]. In our study, treatment of NIH3T3 cells with RA resulted in decreased *LMNA* expression, consistent with previous studies [11,46], a marked increase in nuclear size, and a marked rise in Nestin expression, indicating that RA can induce the differentiation of NIH3T3 cells into neuronal cells. This finding implies that the neuron-specific miR-9 may maintain the neural differentiation and neural fate through suppressing *LMNA* gene expression [47].

Our work thus bridges the gap between materials science and epigenetics, paving the way for next-generation biomaterials that integrate mechanical, chemical, and biological cues to control cell fate transition. By culturing cells on PAA hydrogels with tunable stiffness (0.2 kPa vs. 10 kPa), we demonstrated that PAA hydrogels functionalized with ECM proteins (fibronectin/gelatin) provide a scalable platform to engineer mechanically responsive microenvironments for precise neural induction.

In conclusion, our study establishes a material-based paradigm for controlling cell fate through nucleus-mediated mechano-epigenetic regulation. Our study also identifies a novel approach for inducing fate transition in vitro, based on cell stiffness is positively correlated with Lamin A expression. By inhibiting LMNA expression, neuronal cell fate can be achieved, shedding light on epigenetic mechanisms that underpin cell fate transformation. Importantly, the small-molecule drug RA not only inhibits LMNA expression but also enables the transdifferentiation of readily available cell types into nerve-associated cells. This has marked implications for tissue engineering and regenerative medicine, providing potential seed cells for therapeutic applications.

## Data Availability

The data that support the findings of this study are available from the corresponding authors upon reasonable request.
