# Supplementary material for: Decreasing Lamin A Triggers Cell Fate Transitions through Heterochromatin-Nuclear Periphery Detethering
Source: Biomater Res. 2025 Sep 18;29:0256. doi: 10.34133/bmr.0256 (PMC12444033; doi:10.34133/bmr.0256)
Supplement: Supplementary 1 — Figs. S1 to S8 Table S1 [file bmr.0256.f1.doc]

**Front Matter**

Title

Decreasing lamin A triggers cell fate transitions through heterochromatin-nuclear periphery detethering

**Authors**

Lijuan Sun^1,#^, Yafan Xie^1,#^, Zhaoyan Zuo^1,#^, Jian Liu ^2^, Jiaqi Yang ^1^, Iqra Ali ^1^, Qin Peng^2,*^, Juhui Qiu^1,*^

**Affiliations**

^1^ Key Laboratory for Biorheological Science and Technology of Ministry of Education, State and Local Joint Engineering Laboratory for Vascular Implants, Bioengineering College, Chongqing University, Chongqing 400030, China.

^2^ Institute of Systems and Physical Biology, Shenzhen Bay Laboratory, Shenzhen 518132, China.

#: These authors contributed equally

*Corresponding author: Qin Peng ([pengqin@szbl.ac.cn](mailto:pengqin@szbl.ac.cn)), Juhui Qiu ([jhqiu@cqu.edu.cn](mailto:jhqiu@cqu.edu.cn))

Supplementary Materials

Fig S1-S8

Table S1

**
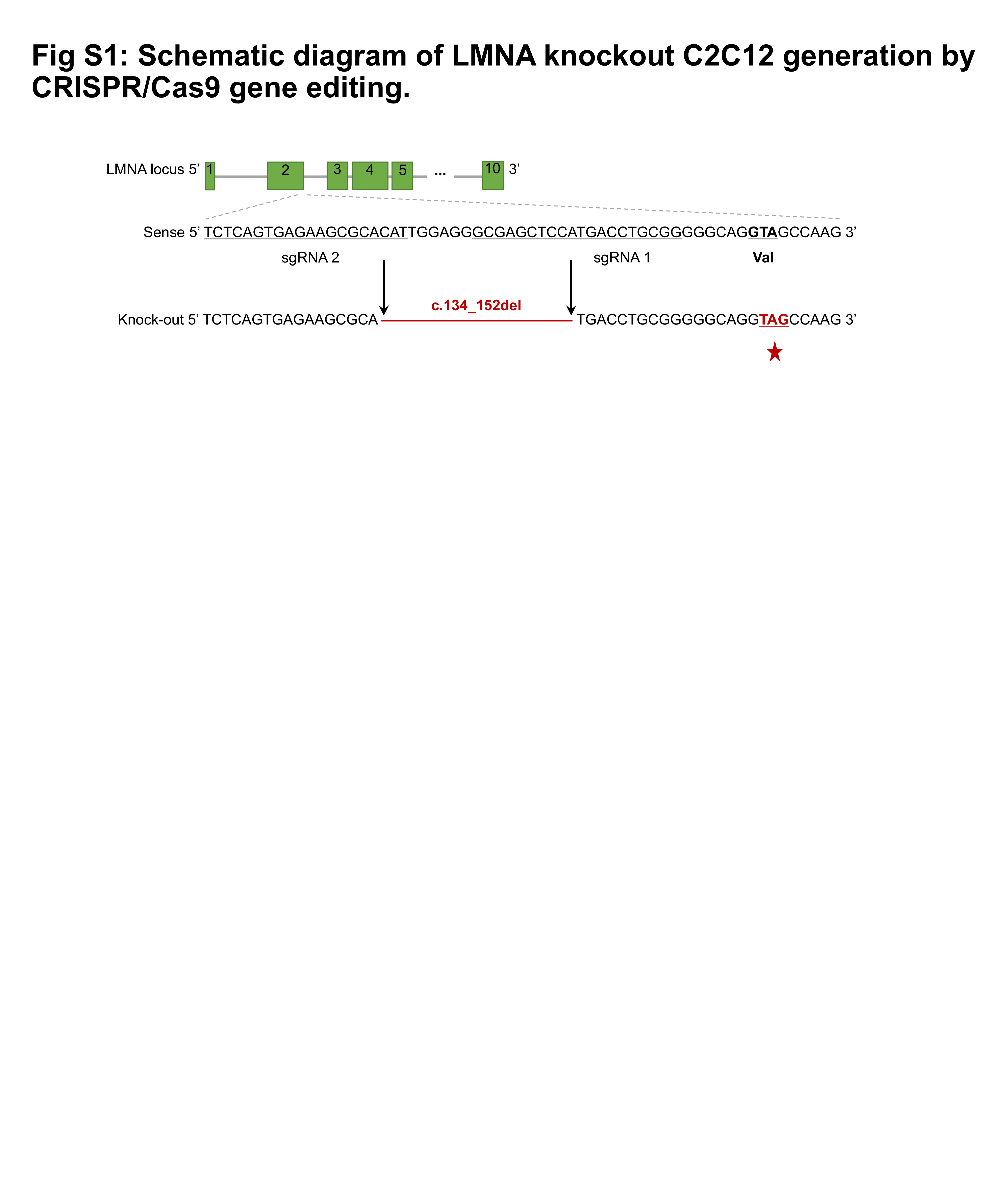
**

**Figure S1: Schematic diagram of *LMNA* KO C2C12 generation by CRISPR/Cas9 gene editing.**


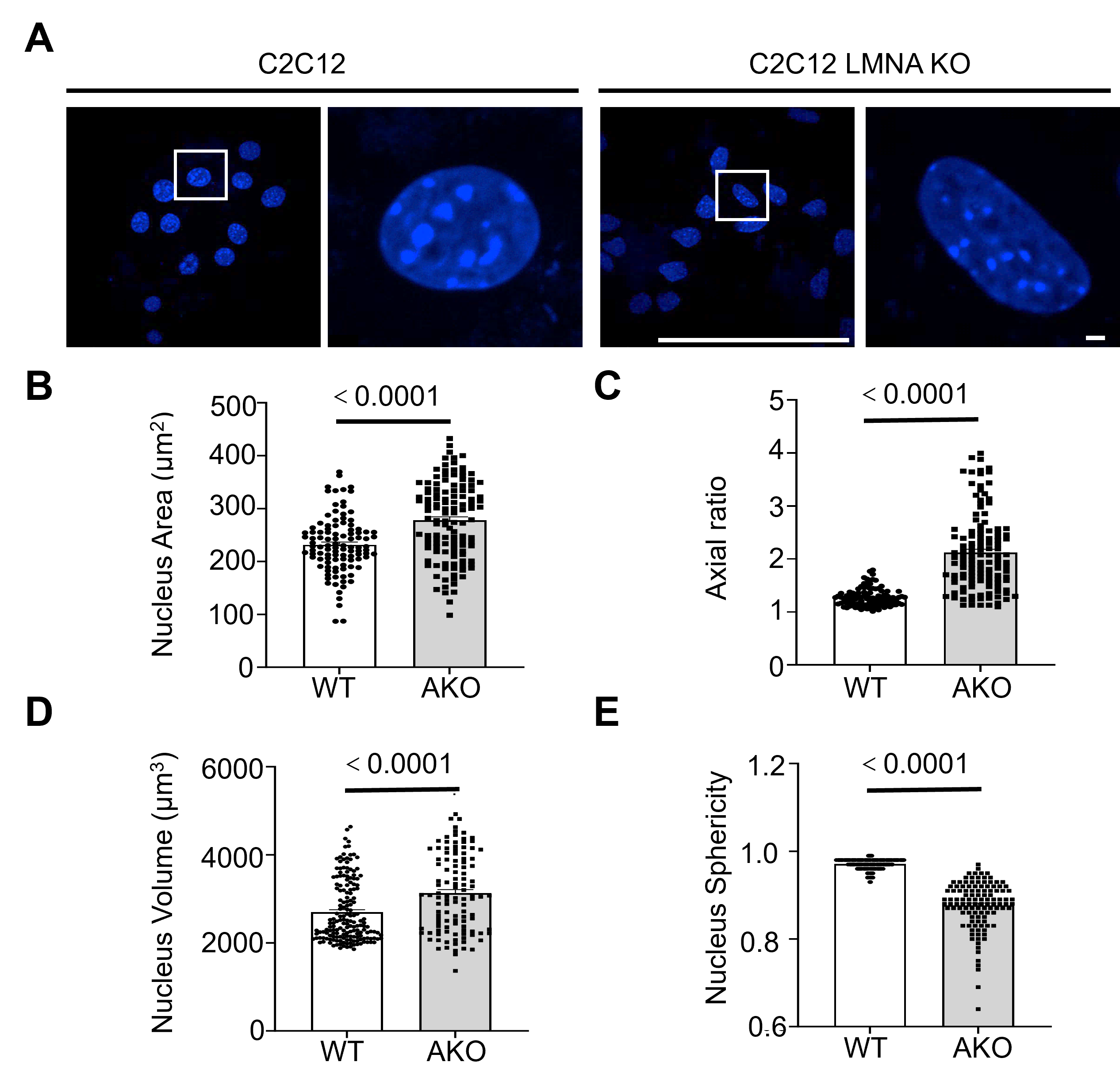


**Figure S2: *LMNA* deletion induces nucleus morphological changes.** (A) Immunofluorescence images; the original image is on the left, and a magnified view of the area within the white box is on the right. Scale bar: 3 µm. (B) Quantitative analysis of nucleus area in C2C12 WT and C2C12 *LMNA* KO cells. n=106, 127. (C) Quantitative analysis of aspect ratio in C2C12 WT and C2C12 *LMNA* KO cells. n=105, 101. (D) Quantitative analysis of nucleus volume in C2C12 WT and C2C12 *LMNA* KO cells. n=173, 112. (E) Quantitative analysis of nucleus sphericity in C2C12 WT and C2C12 *LMNA* KO cells. n=173, 116.Nuclear morphology and statistical analysis of nuclear area and aspect ratio in C2C12 cells post LMNA KO. Scale bar: 3 µm.

**
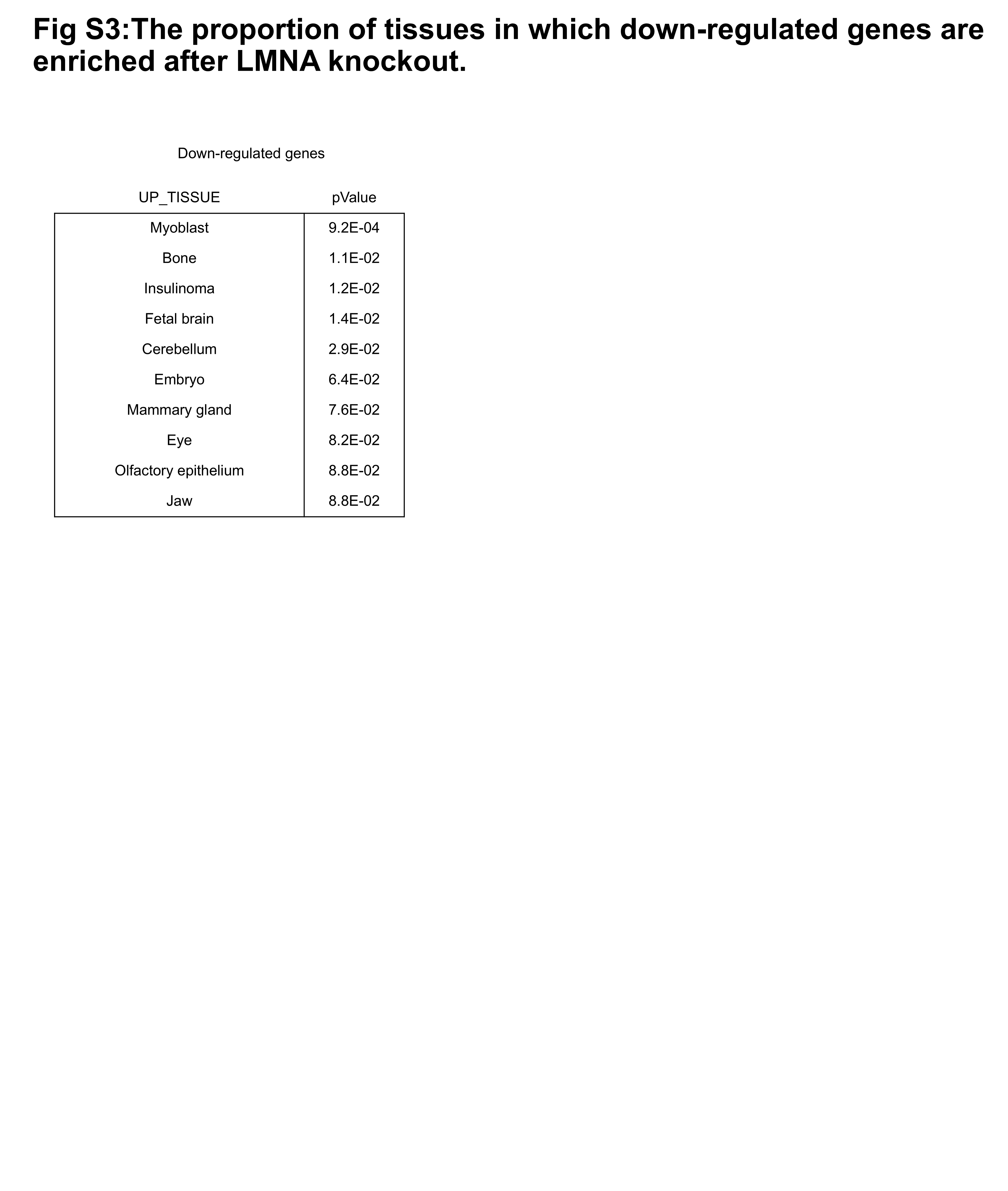
**

**Figure S3:** **The proportion of tissues in which down-regulated genes are enriched after *LMNA* KO.**

**
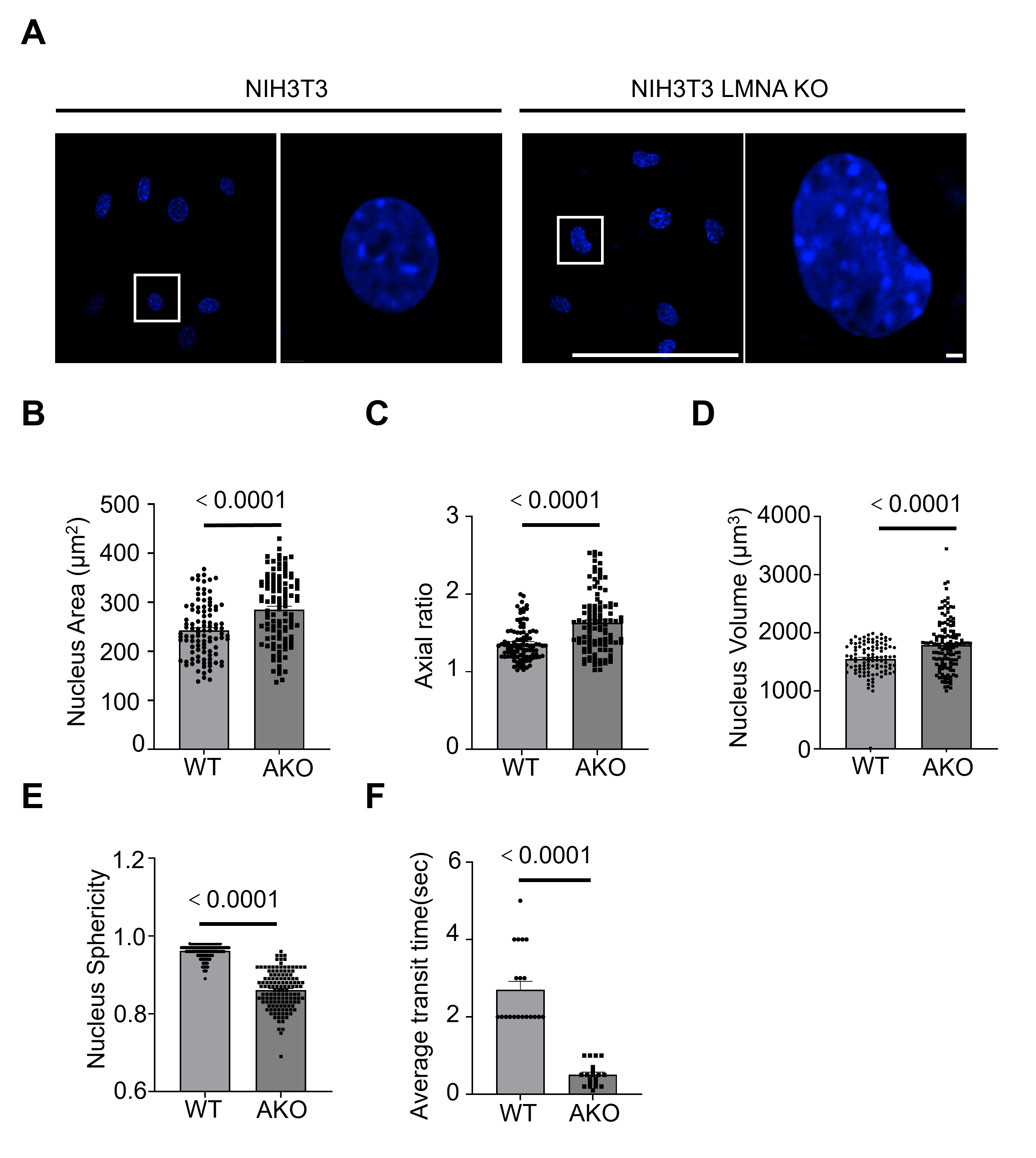
**

**Figure S4: The *LMNA* KO alters the nuclear shape and stiffness of NIH3T3 cells.** (A) Immunofluorescence images; the original image is on the left, and a magnified view of the area within the white box is on the right. Scale bar: 3 µm. (B) Quantitative analysis of nucleusnuclear area in NIH3T3 WT and NIH3T3 *LMNA* KO cells. n=100, 109. (C) Quantitative analysis of the axial ratio in NIH3T3 WT and NIH3T3 *LMNA* KO cells. n=101, 108. (D) Quantitative analysis of the nucleus volume in NIH3T3 WT and NIH3T3 *LMNA* KO cells. n=105, 133. (E) Quantitative analysis of the nucleus sphericity in NIH3T3 WT and NIH3T3 *LMNA* KO cells. n=159, 133. (F) Time taken for each cell to pass through the microfluidic channel pore. n=20.

**
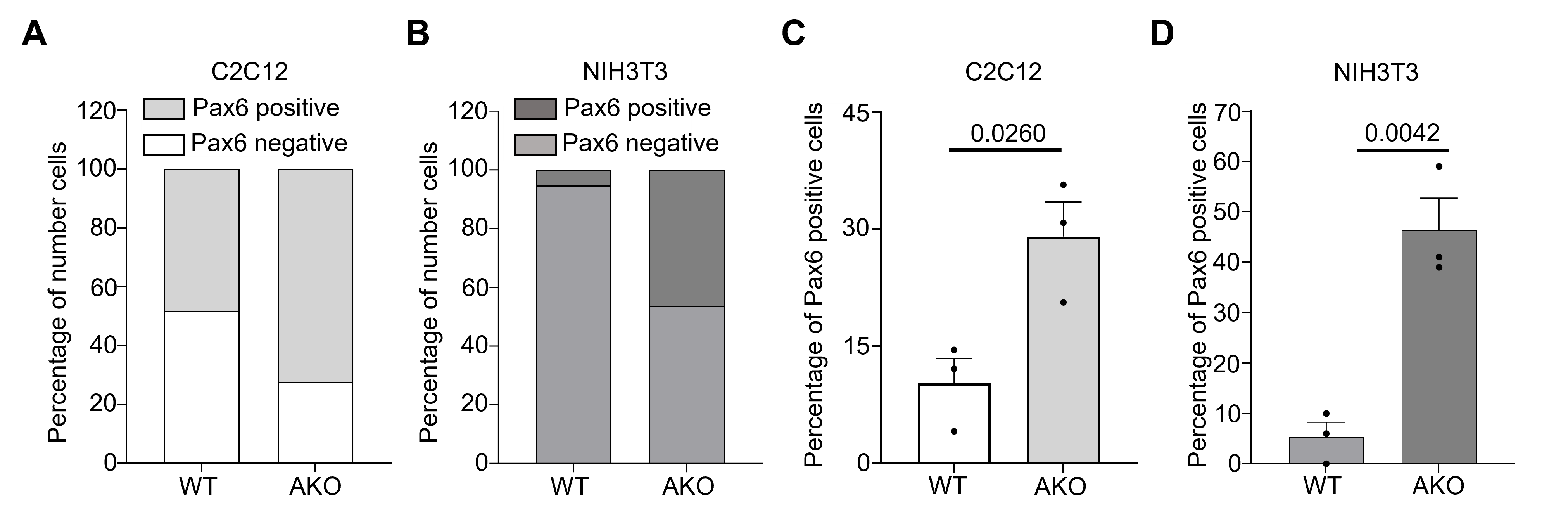
**

**Figure S5: Percentage of Pax6 positive and negative cells in cells after *LMNA* KO.** Percentage of Pax6-positive and -negative cells in C2C12 WT and C2C12 *LMNA* KO. (B) Percentage of Pax6-positive and -negative cells in NIH3T3 WT and NIH3T3 *LMNA* KO cells. (C) Percentage of Pax6-positive cells in C2C12 WT and C2C12 *LMNA* KO. (D) Percentage of Pax6-positive cells in NIH3T3 WT and NIH3T3 *LMNA* KO cells. All experiments were repeated three times.

**
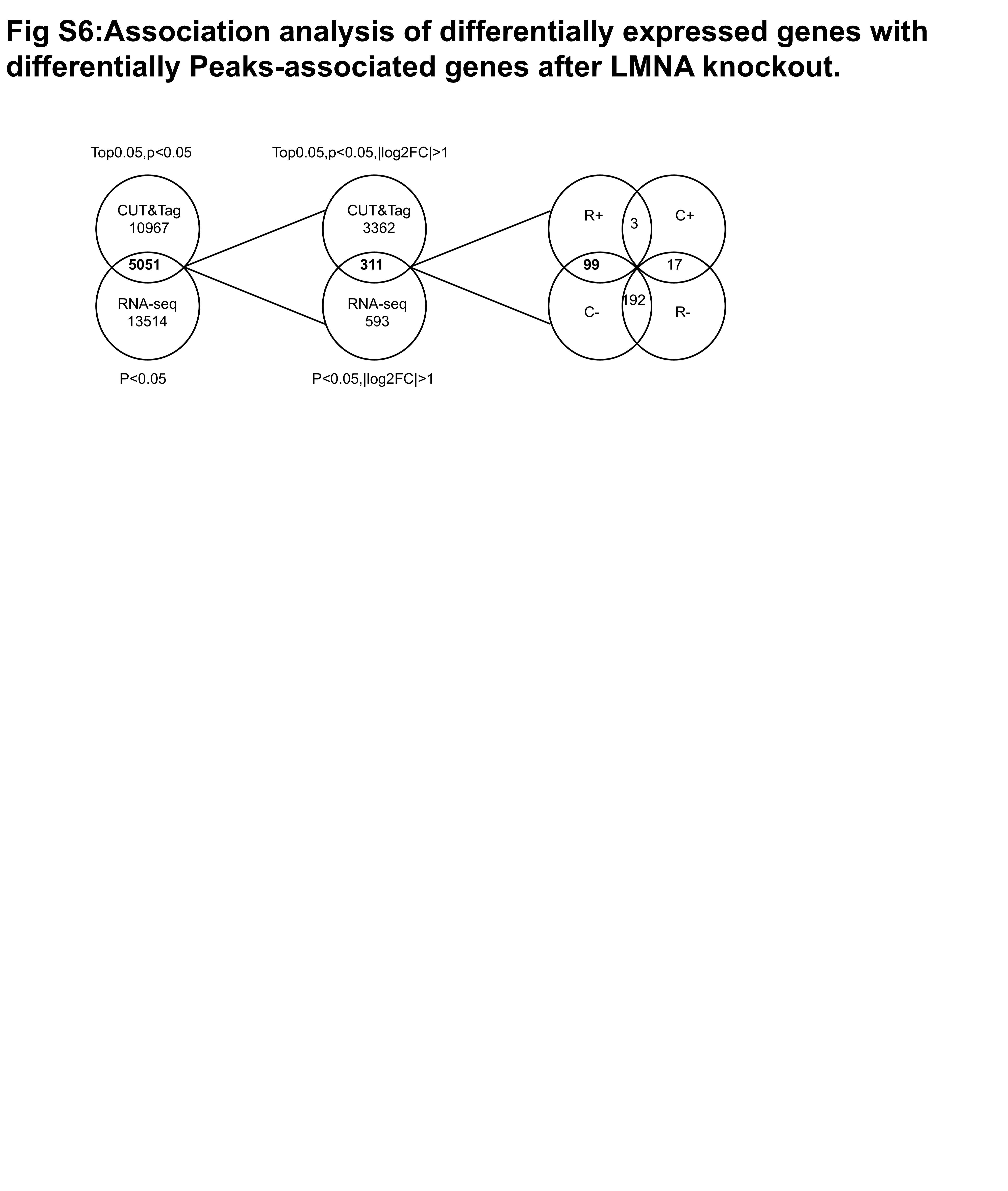
**

**Figure S6: Synapsis analysis of differentially expressed genes and differentially peaks associated genes after *LMNA* KO.**

**
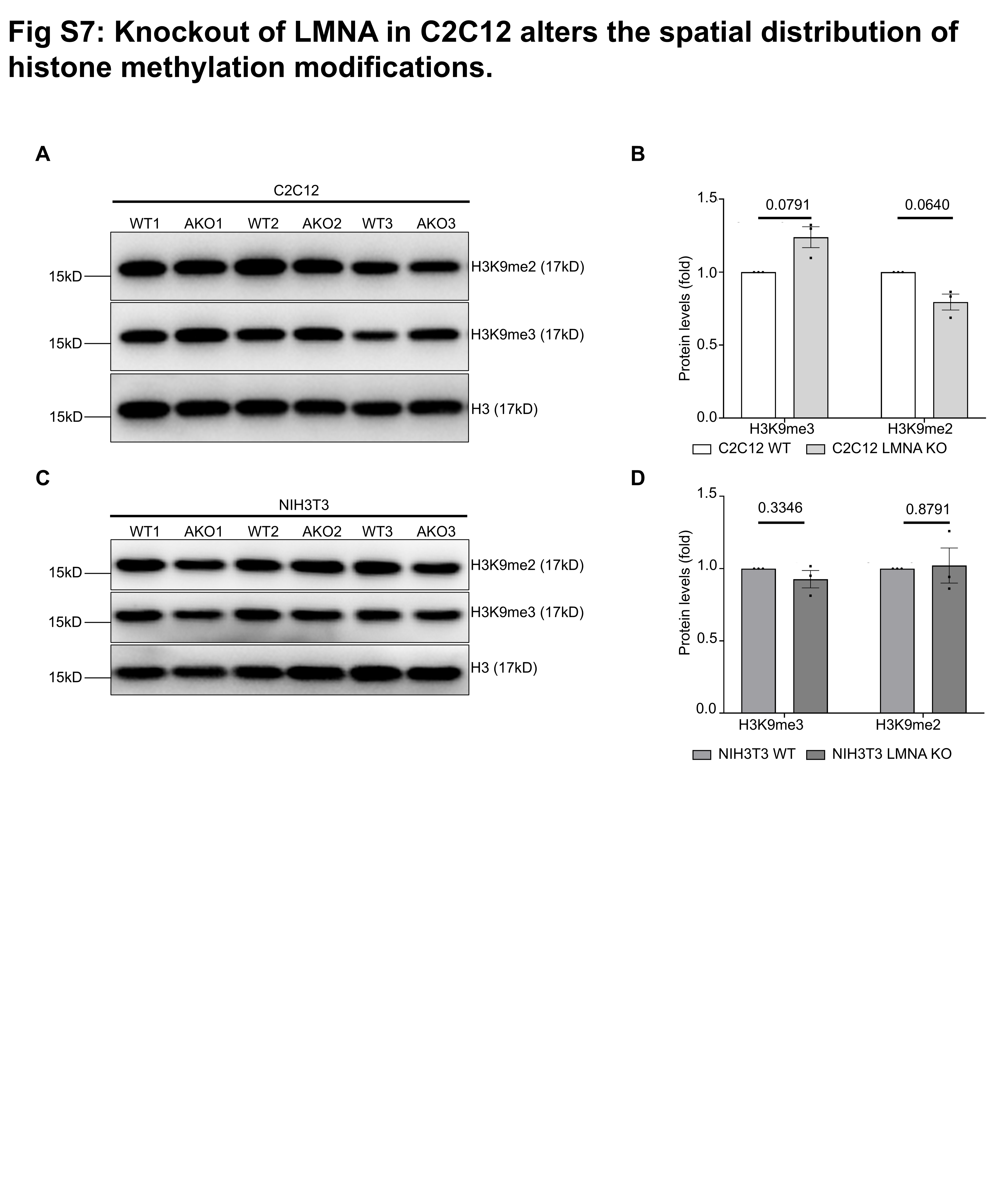
**

**Figure S7:** **The KO of *LMNA* in cell does not alter the levels of histone methylation modification.** (A) WB analysis of H3K9me3 and H3K9me2 levels in C2C12 WT and C2C12 *LMNA* KO. (B) Quantitative analysis of H3K9me3 and H3K9me2 levels in C2C12 WT and C2C12 *LMNA* KO. (C) WB analysis of H3K9me3 and H3K9me2 levels in NIH3T3 WT and NIH3T3 *LMNA* KO. (D) Quantitative analysis of H3K9me3 and H3K9me2 levels in NIH3T3 WT and NIH3T3 *LMNA* KO. All experiments were performed in 3 replicates.

**
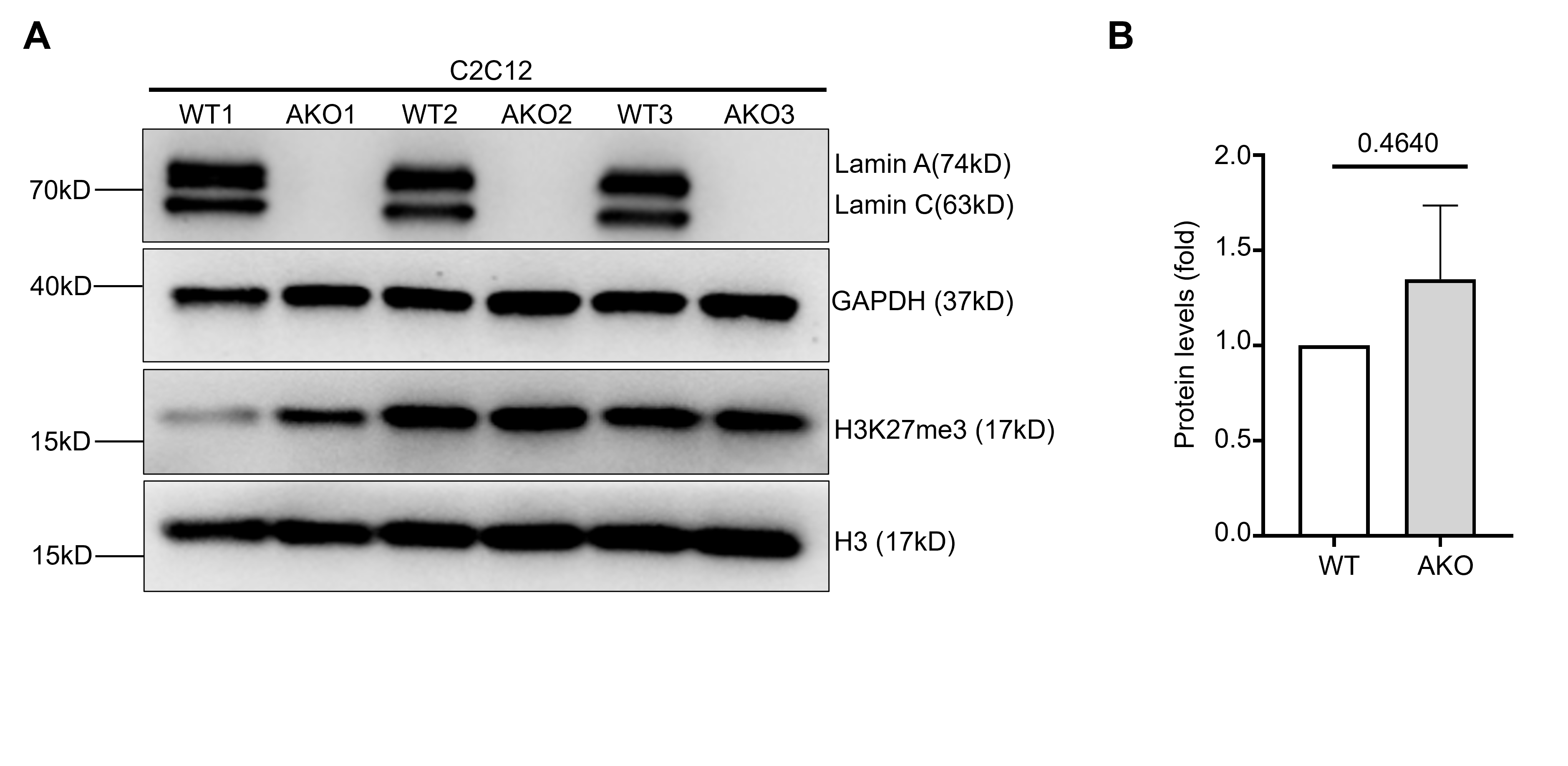
**

**Figure S8:** **The KO of *LMNA* in cell does not alter the levels of H3K27me3.** (A) WB analysis of H3K27me3 levels in C2C12 WT and C2C12 *LMNA* KO. (B) Quantitative analysis of H3K27me3 levels in C2C12 WT and C2C12 *LMNA* KO. All experiments were performed in 3 replicates.

**Table S1. Biological process of gene enrichment in C2C12 upregulation of transcriptional expression after *LMNA*** KO

| Biological Processes | Gene |
| --- | --- |
| synapse assembly | CADM1, CDH2, FLRT3, PCDHB16, CACNA1A, VPS35, BSN, ZDHHC2, OBSL1, CBLN1 |
| axon guidance | ROBO2, SEMA5A, RTN4R, EPHB6, EPHA4, SEMA4D, NOG, WNT5A, SEMA3G, PAX6, UNC5C, SEMA3E, SEMA3F, ABLIM1, LHX2, FLRT3, KIF5A |
| nervous system development | SEMA5A, ROBO2, CXCR4, SEMA3E, PTPRF, ADGRG1, ZIC2, ATOH8, PCDHAC2, NKX2-2, TFAP2A, EPHA4, MBNL1, SEMA4D, NTRK3, LHFPL4, GFRA1, ZIC5, POU4F1, DCLK1, GFRA4, LHX2, LHX1, SARM1, RAPGEF2, GAS7 |
| negative regulation of axon extension involved in axon guidance | SEMA5A, SEMA4D, WNT5A, SEMA3G, SEMA3E, SEMA3F |
| telencephalon regionalization | BMP4, LHX2, SIX3, PAX6 |
| telencephalon development | BMP4, CDH2, LHX2, LHX1, SIX3, RPGRIP1L |
| negative regulation of neuron apoptotic process | TFAP2A, NFIX, ANGPT1, PCDHGC4, CACNA1A, SEMA3E, MT1, POU4F1, AGT, IL27RA, PRDX2, UCP2, HMOX1, NOS1, RETREG1 |
| synaptic membrane adhesion | PTPRD, LRFN3, CADM1, FLRT3, PTPRF, PCDH17 |
| cell differentiation | SEMA5A, ROBO2, TCF23, LRP4, SEMA3E, LGALS3, ADGRG1, RHOX5, ZIC2, ATOH8, NKX2-5, JUNB, WNT4, NKX2-2, JAG2, SMAD1, TCF7L2, TFAP2C, ANGPT2, SEMA4D, CADM1, ANGPT1, SYK, NTRK3, STYK1, NOG, WNT5A, CAVIN4, SIAH1A, SMAD9, PAX6, ZIC5, KAZALD1, HOPX, DCLK1, RBL2, BMP4, NUDT21, SFRP1, SFRP2, ID2, LHX1, PMP22, SARM1, ID3, RAPGEF2, GAS7 |
|  |  |

**Table S1. Biological process of gene enrichment in C2C12 upregulation of transcriptional expression after *LMNA* KO** (continued)

| Biological Processes | Gene |
| --- | --- |
| neural tube closure | TFAP2A, BMP4, SFRP1, SFRP2, ZIC2, LHX2, WNT5A, NOG, ZIC5, PRKACA |
| brain development | ROBO2, NFIX, CADM1, NOG, CXCR4, PAX6, UNC5C, RPGRIP1L, DCLK1, PYGO2, ADGRG1, CDH2, LHX2, COL4A1, BGLAP2, SIX3, SLC17A7 |
| astrocyte differentiation | NFIX, MYCN, ID2, PAX6, NKX2-2 |
| neuron differentiation | NFIX, WNT5A, PAX6, GFRA1, WNT9A, POU4F1, PTPRD, LHX2, LHX1, ATOH8, ID3, IER2, NKX2-2, WNT4, GAS7 |
| olfactory bulb interneuron development | ROBO2, WNT5A, ATF5 |
| negative regulation of neurotrophin TRK receptor signaling pathway | AGTR2, PTPRF, AGT |
| pituitary gland development | BMP4, TCF7L2, NOG, SIX3, PAX6 |
| neurotransmitter receptor localization to postsynaptic specialization membrane | LHFPL4, NPTX1, NPTXR, RAPSN |
